# Content validity of patient-reported measures evaluating experiences of the quality of transitions in healthcare settings—a scoping review

**DOI:** 10.1186/s12913-024-11298-0

**Published:** 2024-07-22

**Authors:** Sisse Walløe, Stine Gundtoft Roikjær, Sebrina Maj-Britt Hansen, Graziella Zangger, Sofie Rath Mortensen, Christoffer Bruun Korfitsen, Charlotte Simonÿ, Henrik Hein Lauridsen, Lars Morsø

**Affiliations:** 1 Department of Clinical Research, Research Unit OPEN, University of Southern Denmark, Odense, Denmark; 2 Department of Physio- and Occupational Therapy, Research- and Implmentation Unit PROgrez, Næstved-Slagelse-Ringsted Hospitals, Region Zealand, Denmark; 3 Department of Neurology, Center for Neurological Research, Næstved-Slagelse-Ringsted Hospitals, Region Zealand, Denmark; 4 Department of Health, Institute of Regional Health Research, University of Southern Denmark, Odense, Denmark; 5 Department of Sports Science and Clinical Biomechanics, Research Unit for Musculoskeletal Function and Physiotherapy, University of Southern Denmark, Odense, Denmark; 6 Department of Sports Science and Clinical Biomechanics, Research Unit for Exercise Epidemiology, University of Southern Denmark, Odense, Denmark; 7 Department of Clinical Research, Cochrane Denmark & Centre for Evidence-Based Medicine Odense (CEBMO), University of Southern Denmark, Odense, Denmark; 8 Department of Sports Science and Clinical Biomechanics, University of Southern Denmark, Odense, Denmark

**Keywords:** Healthcare transitions, Patient-Reported Experience Measure, Patient Experience, Quality, Scoping Review

## Abstract

No reviews so far have been conducted to define the constructs of patient-experienced quality in healthcare transitions or to identify existing generic measures of patients’ experience of the quality within healthcare transitions. Our aim was to identify domains relevant for people experiencing healthcare transitions when evaluating the quality of care they have received, map the comprehensiveness of existing patient-reported experience measures (PREM), and evaluate the PREMs’ content validity. The method was guided by the Joanna Briggs Institutes’ guidance for scoping reviews. The search was performed on 07 December 2021 and updated 27 May 2024, in the electronic databases Medline (Ovid), Embase (Ovid), and Cinahl (EBSCO). The search identified 20,422 publications, and 190 studies were included for review. We identified 30 PREMs assessing at least one aspect of adults’ experience of transitions in healthcare. Summarising the content, we consider a model with two domains, organisational and human-relational, likely to be adequate. However, a more comprehensive analysis and adequate definition of the construct is needed. None of the PREMs were considered content valid.

**Supplementary Information:**

The online version contains supplementary material available at 10.1186/s12913-024-11298-0.

## Background

Care management encompasses evaluating individuals' needs and coordinating healthcare services; however, patients struggle with consistency and clarity in care management [1]. Patients’ ability to navigate healthcare systems has implications for the outcome of their treatment [2, 3] such as functional ability [4], illness management [5], disease relapse [6], and quality of life [7], and patients experience navigation of healthcare services as burdensome [8]. Furthermore, healthcare structures which are difficult to navigate accentuate existing social inequlities in healthcare [9] and people with poor health literacy are at higher risk of poor quality of care in healthcare transitons [10–12]. Because patients’ experiences are associated with clinical effectiveness and safety [13], assessing patients’ experiences with transitions in healthcare is important in determining the quality of healthcare delivery. Patient-reported experience measures (PREM) are intended to be reliable measures of the quality of healthcare services from patients’ perspectives and may support evaluation of the effectiveness, safety, and efficiency of healthcare provision [14, 15]. However, validity and reliability criteria of PREMs are often indadequatly investigated before clinical application of the PREMs, potentially leading to issues of responsiveness when applied in clinical trials [16].

Several reviews of instruments measuring patient experience of quality in healthcare transitions have been published [17–22]. In the systematic review by Berbee et al. [23] they aimed to identify and select an appropriate instrument for measuring the quality of integrated care for patients experiencing chronic illness, but identified no patient-reported experience measure that was comprehensive or relevant according to focus group interviews with the patients [17]. Concordantly, in a systematic review for evaluating patient satisfaction in healthcare settings integrating behavioural and medical health services, Black et al. [18] found that no PREM comprehensively captured all relevant aspects of the integrated healthcare experience [18]. In contrast, Weaver et al. [19] reviewed concepts, models, and instruments for understanding care continuity in mental health services and suggested a PREM suitable for self-reporting experiences with mental healthcare [19]. In 2011 Fiscella et al. [20] published a consensus of domains and measures suitable for patient-reported assessment of cancer navigation but also called for an instrument that covered all relevant topics [20]. Likewise, McMurray et al. [21] identified 25 instruments to assess patients’ experience of rehabilitation services, but none comprehensively measured patient experience across the rehabilitative care continuum [21]. Following this, Quinn et al. [22] concluded that no instruments adequately assessed veterans’ experience with access and coordination across healthcare settings [22]. No reviews so far have identified a generic PREM that comprehensively measures patients’ experience with the quality of healthcare transitions [17–22, 24, 25]. Therefore, there is a need to identify adequate concepts and measures that can target patient experienced transitions in healthcare.

The “COnsensus-based Standards for the selection of health Measurement Instruments (COSMIN)” initiative was founded by a group of reasearcher with a mission to improve the quality of measurement of health outcomes [26]. The COSMIN group argues that content validity is the most important aspect of patient-reported measures [27]. Content validity refers to a patient-reported measure's relevance, comprehensiveness, and comprehensibility [28]. In other words, to evaluate whether the instrument provides an adequate reflection of the construct to be measured in the context [27]. Although there is some consensus on defining patient experience of healthcare transitions as a multidimensional concept consisting of human-relational and system factors, the conceptualizations found in existing reviews still lack clarity [17–22, 24, 25].

The overall scope of this review was to explore and define domains of the concept of patient-experienced quality in cross-sectoral care for generic patient populations. Further, to map existing methods for generically assessing the quality of transitions in healthcare settings (e.g. between municipality, general practitioner, and hospital). To achieve this, our objectives were:

1. What domains are considered relevant to measure for patients’ experiencing healthcare transitions when evaluating the quality of care they have received?
2. What existing patient-reported experience measures attempt to measure patients’ experience of transitions in healthcare settings?
3. Are any existing patient-reported experience measures adequate (relevant and comprehensive) reflections of patients’ experience of transitions in healthcare settings?

## Methods

The method of this scoping review followed the Joanna Briggs Institutes’ (JBI) guidance for scoping reviews [29]. The article was outlined following JBI guidelines [29] supported by the Preferred Reporting Items for Systematic Reviews and Meta-Analyses extension for Scoping Reviews (PRISMA-ScR) Checklist [30]. A pre-registered protocol, including aims, search strategies, and amendments made during the iterative review process [29], is available via Open Science Framework (OSF) [31]. The research question was defined using the SPIDER-model (i.e., Setting, Phenomenon of Interest, Design, Evaluation, Research) [32]. The research question encompassed five concepts with predefined definitions and in- and exclusion criteria to ensure identification of eligible studies (Table 1).

**Table 1:** Research Question Defined by the SPIDER^a^-Model

		Concepts	Inclusion criteria	Exclusion criteria
S	Setting	Transitions in healthcare settings	Patient transition between at least 2 healthcare settings (municipality, GP, hospital)	Single settings such as “In the primary care setting, at the hospital etc.”
PI	Phenomenon of Interest	Patient-experienced quality	Patients’ experiences were accounted for or assessed	Only healthcare personnel or relatives’ experiences were assessed
D	Design	Qualitative and quantitative research designs	Psychometric studies, qualitative studies, quantitative studies, syntheses/reviews	Feasibility studies, study protocols, reports
E	Evaluation	PREMs^b^, patient accounts, narratives, attitudes, perspectives, and experiences of quality	Patient experience of healthcare provision [21, 33]	Patient satisfaction [34]
R	Research Type	Published, peer-reviewed research reporting data	Peer-reviewed, published studies	Conference abstracts and meeting notes

^a^Setting, Phenomenon of Interest, Design, Evaluation, Research type

^b^Patient-Reported Experience Measure

### Search

The search was performed on 07 December 2021 and updated 27 May 2024. The electronic databases Medline (Ovid), Embase (Ovid), and Cinahl (EBSCO) were chosen because they cover multiple research areas within healthcare. The search was developed in Embase and translated to Medline and Cinahl as recommended [35]. For the phenomenon of interest, we identified the Medical Subject Heading (MeSH) term “Patient Satisfaction”. Although we excluded studies reporting on patient satisfaction rather than patient experience, the terms have been used interchangeably, [21] and patient satisfaction thus seemed necessary to include in our search strategy. As the focus on patient's experiences of coherent care seemed to have emerged around late 1990 and early 2000, [36] we searched for literature from 2000. The search strategy was developed from the predefined definitions and criteria with guidance from research librarians. An example of the search can be seen in Table 2 (see full search strategy in Supplementary material 1).

**Table 2:** Example of search syntax

	MEDLINE Ovid (Ovid MEDLINE(R) ALL)
1	"continuity of patient care"/ or patient discharge/ or patient handoff/ or patient transfer/ or retention in care/ or transitional care/
2	*"Delivery of Health Care, Integrated"/
3	(care adj2 continu*).ab,kf,ti
4	(care adj2 across adj5 sectors).ab,kf,ti
5	(care adj2 ?cross adj5 sector*).ab,kf,ti
6	(inter* adj2 sector* adj2 care).ab,kf,ti
7	(integrat* adj care).ab,kf,ti
8	(transition* adj2 care).ab,kf,ti
9	(coordinat* adj3 care).ab,kf,ti
10	1 or 2 or 3 or 4 or 5 or 6 or 7 or 8 or 9
11	exp Patient Satisfaction/
12	(patient* adj1 experience*).ab,ti
13	(patient* adj1 perspective*).ab,ti
14	(patient* adj2 view*).ab,kf,ti
15	(patient* adj2 attitude*).ab,kf,ti
16	(patient* adj2 satisf*).ab,kf,ti
17	(patient* adj2 involvement*).ab,kf,ti
18	(user* adj2 perspective*).ab,kf,ti
19	(user* adj2 view*).ab,kf,ti
20	(user* adj2 involvement*).ab,kf,ti
21	(user* adj2 attitude*).ab,kf,ti
22	(user* adj2 satisf*).ab,kf,ti
23	(user* adj2 involvement*).ab,kf,ti
24	(people* adj1 experience*).ab,ti
25	(people* adj1 perspective*).ab,ti
26	(people* adj2 view*).ab,kf,ti
27	(people* adj2 attitude*).ab,kf,ti
28	(people* adj2 satisf*).ab,kf,ti
29	(people* adj2 involvement*).ab,kf,ti
30	11 or 12 or 13 or 14 or 15 or 16 or 17 or 18 or 19 or 20 or 21 or 22 or 23 or 24 or 25 or 26 or 27 or 28 or 29
31	10 and 30
32	Limit 31 to yr = ”2000-Current”

### Selection of sources of evidence

Covidence (Veritas Health Innovation, Melbourne, Australia) was used to manage the duplication and screening process [37]. All studies were screened by two independent reviewers, and a total of seven reviewers participated in the screening process. To ensure calibration of the screening process, a consensus meeting was arranged at the beginning of the process as in rapid reviews [38]. During the calibration sessions, in- and exclusion criteria were specified further than the a-priori defined criteria (Supplementary material 2).

In the full-text screening process, we experienced a larger number of articles than expected. In order to focus this review on the a priori defined aims, we decided to; I) report on intervention studies in an independent review, II) exclude mixed-methods studies and original qualitative studies, III) synthesize dimensions found in the included syntheses and reviews rather than report on the original studies. We decided to include syntheses and reviews because reports on qualitative studies were frequent, and relevant themes for patient-experienced quality of healthcare transitions had already been mapped in these meta-syntheses, integrative reviews, or scoping reviews.

### Data charting process

A priori-defined data extraction templates were used and are available at OSF [39]. The data charting was done by one author (SW). The first 10 data extractions were validated independently by a research assistant (NH). The data charting table and process were adapted following the pilot extraction. The final data extraction tables are available in Supplementary materials 3 and 4.

### Critical appraisal of individual sources of evidence

Although the JBI guidelines for scoping reviews do not warrant critical appraisal, [40] we critically appraised a selection of the most comprehensive PREMs according to the COSMIN Risk of Bias checklist for systematic reviews of Patient-Reported Outcome Measures [27, 41, 42] to assess content validity [27] (relevance, comprehensiveness, and comprehensibility) from patients’ perspectives (See Supplementary material 5 for checklist). The critical assessment was done independently by two reviewers (SW, LM), and conflicts were discussed until a consensus was reached. When PREMs were mentioned in included publications but not available in the publication, references were followed to the original publications on that PREM.

### Synthesis of results

**Fig. 1:**
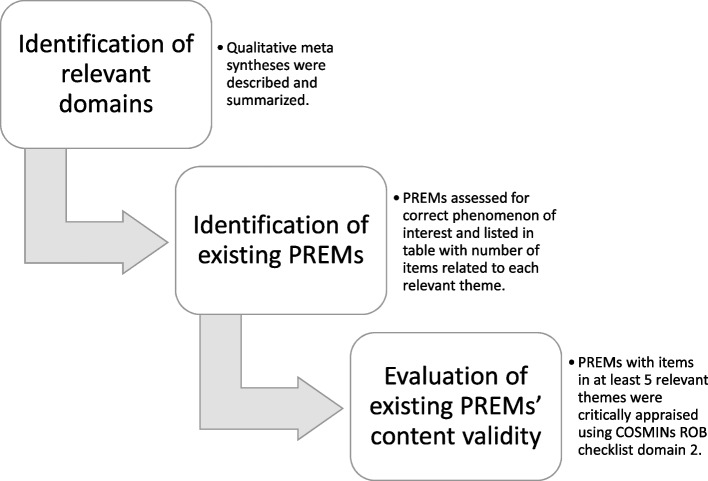
Data Synthesis Process

We synthesized the data by; I) Summarizing themes identified in the qualitative syntheses and identifying relevant domains (Supplementary material 3 and Fig. 3); II) Identifying PREMs, assessing the PREMs phenomenon of interest, and categorizing and listing the items of the relevant PREMs to assess comprehensiveness (Supplementary materials 4, 6 and Table 3); III) Assessing the content validity of the most comprehensive PREMs (items related to five or more themes) (Table 3). The process of synthesizing data is also described in Fig. 1.

**Table 3:** Comprehensiveness and Content Validity of Existing PREMs for Assessment of Patients’ Experiences of the Quality of Transitions in Healthcare Settings

	Themes relevant for measuring patient-experienced quality in healthcare transitions as identified in qualitative literature (objective 1) – *n* = number of items of PREM^a^ for the named theme													
PREM	Patient-centeredness and individualized care	Effectiveness	Efficiency, coordination, and management	Timeliness	Equity	Caring attitude and compassion	Navigation, access, and availability	Responsibility	Relational Continuity and relationship	Informational continuity	Communication and education	Caretakers and relatives	Articles referencing this PREM	Content Validity
ACSS-MH Alberta Continuity of Services Scale—Mental Health	6	3	9	2			8	4	5	2		2	Joyce 2010 [43], Fernandes 2020 [24], Weaver 2017 [19]	Adequate
CCAENA Questionnaire of Continuity between Care Levels	2		7	2			2		6	4	4	4	Aller 2013 [44], Vargas 2013 [45], Karam 2019 [46]	Inadequate
CCRQ Client-Centered Rehabilitation Questionnaire	8	4	2			2	4		1		6	4	McMurray 2016 [47]	Doubtful
CONTINU-UM Continuity of care – User Measure	5	1	3	1			4		2		1		Rose 2009 [48], Fernandes 2020 [24], Weaver 2017 [19], Sweeney 2012 [49]	Doubtful
CPCI Components of Primary Care Index	3	1	3					1	6	2	2		Quinn 2017 [22]	Inadequate
CPCQ Client Perception of Continuity Questionnaire	7	3	6	1			1	1		2	1	2	McGuiness 2003 [50], Quinn 2017 [22]	Inadequate
CQI Consumer Quality Index (Continuum of Care)		1	4	2			1		2	4			Berendsen 2009 [51], Willems 2013 [52], Kollen 2011 [53]	Doubtful
CTM Care Transition Measure	3		2								10		Acosta 2017 [54], Bakshi 2012 [55], Coleman 2005 [56], Shadmi 2009 [57], Mosallam 2014 [58], Hod 2020 [59]	Inadequate
DCCS Diabetes Continuity of Care Scale	7	6	4	6	3		8		5	3	4	1	Dolovich 2004 [60]	Doubtful
ECC Experienced Continuity of Care	2	2	5	2		1			2	4	1		Gulliford 2006 [61], Fillion 2012 [62]	Inadequate
HCCQ Heart Continuity of Care Questionnaire	1	2	5				2	2		6	13		Hadjistavropoulos 2004 [63], Hadjistavropoulos 2008 [64], Kowalyk 2004 [65], Valaker 2019 [66], Riley 2007 [67]	Inadequate
NCQ Nijmegen continuity of care questionnaire	3		13					2	5	6			Cohen Castel 2018 [68], Hetlevik 2017 [69], Uijen 2012 [70], Fernandes 2020 [24], Weaver 2017 [19], Hopstaken 2021 [71], den Herder-van der Eerden 2018 [72]	Doubtful
P3CEQ Person centred coordinated experience questionnaire	4	2	2			1		1		1	2		Lloyd 2019 [73], Sugavanam 2018 [74]	Adequate
PACIC Patient Assessment of Chronic Illness Care	10	2	2			1				2	3		Drewes 2012 [75, 76], Berbee 2009 [23], Fernandes 2020 [24], Quinn 2017 [22], Cramm 2013 [77, 78], Bower 2018 [79]	Inadequate
PECQ Patient Experienced Continuity of care Questionnaire	4	1	2			1	2	1	1	2	5	1	Ljungholm 2024 [80]	Doubtful
PCAS The Primary Care Assessment Survey	4	2	2			1		1		1	11		Quinn 2017 [22], O'Malley 2009 [81]	Inadequate
PCCQ Patient Continuity of Care Questionnaire	7		6				5		6	2	12	2	Fillion 2012 [62], Quinn 2017 [22], Sisler 2012 [82], Carneiro 2016 [83]	Inadequate
PEICS Patient Experience of Integrated Care Scale	3	2	2				4				4	2	Joober 2018 [84]	Adequate
PPCMC Patient Percieved Continuity from Multiple Clinicians		1	8					2		6	6		Haggerty 2012 [85], Quinn 2017 [22], Tremblay 2017 [86], Breton 2012 [87]	Doubtful
PPIC Patient Perceptions of Integrated Care	3		6	2			1	1		6	4		Kiang 2013 [88], Mohr 2019 [89], Fryer 2016 [90], Benzer 2019 [91]	Doubtful
SHEP Survey of Healthcare Experiences of Patients	5		1	2		2					5		Black 2021 [18], Fernandes 2020 [24], Quinn 2017 [22]	Inadequate
VANCOSS Veteran Affairs National Outpatient Customer Satisfaction	7		4	7		2	7			4	8	1	Black 2021 [18], Quinn 2017 [22]	Inadequate

^a^Patient-reported experience measure

## Results

**Fig. 2:**
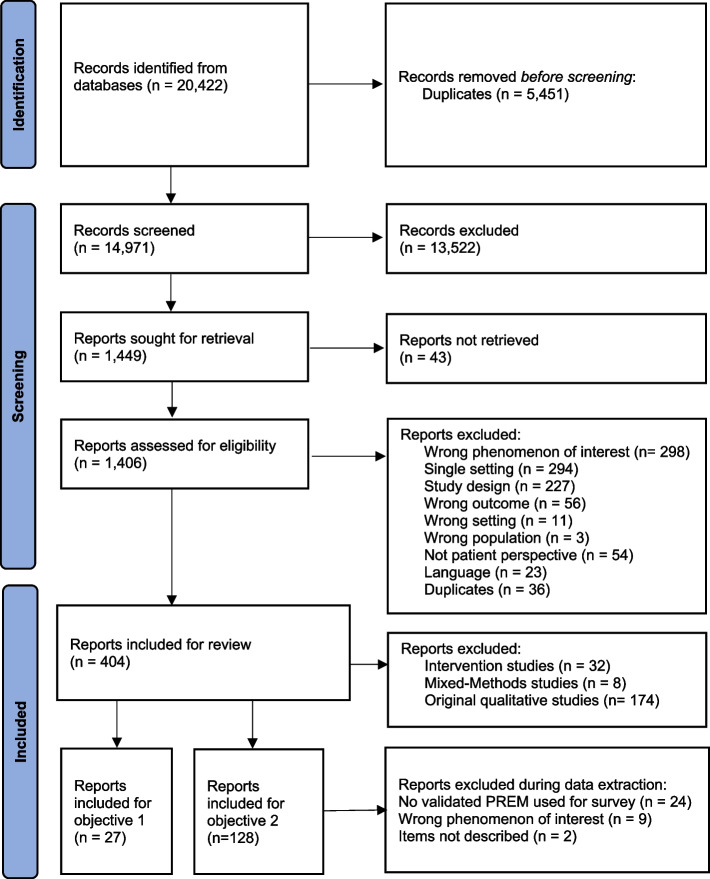
PRISMA Flowchart

The search identified 20,422 records (Fig. 2), and 190 reports were included after the screening and selection process (Fig. 2). Reviewers had 70%-93% agreement. In addition to the regular selection process, first author SW divided the reports according to objectives 1 (27 reports) and 2 (163 reports, 35 reports excluded during data extraction, leaving 128 reports for inclusion). This was done because the scientific methods for reports to answer objective 1 needed to be qualitative, and the methods relevant for answering objective 2 needed to be quantitative observational, development, or validation reports.

### Relevant domains (Results for Objective 1)

**Fig. 3:**
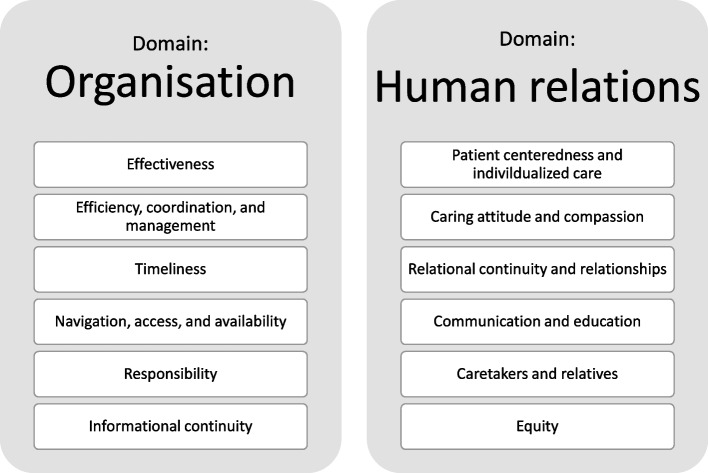
Domains, Themes, and Items Relevant for Assessing Patients’ Experiences of Pathways Across Healthcare Settings

For objective one, we included 27 reports (Supplementary material 7), [47, 92–117] which included: 10 (37%) systematic reviews, [47, 96, 98, 99, 102, 105, 107, 108, 110, 112] seven (26%) scoping reviews, [94, 100, 103, 109, 111, 114, 115] three (11%) meta syntheses, [92, 101, 113] three (11%) integrated reviews, [95, 97, 104] two (7%) narrative reviews, [117, 118] two (7%) qualitative reviews, [106, 116] that provided information on relevant domains and/or themes for assessing patients´ experiences with care across healthcare settings. The number of identified themes ranged from two [94] to 13, [111] with most articles reporting three themes [96, 101, 113, 114, 117]. When summarizing and describing themes from the included reports, we found that the themes could be organized in two distinct domains; I) A system/organizational domain; II) A human-relational domain. Each of these domains encompassed six themes; thus, we identified 12 relevant themes as illustrated in Fig. 3.

The organizational domain included themes that had to do with delivery of healthcare services such as timeliness and efficiency [104, 106]. The human-relational domain was more about how services were delivered [107, 108]. We found that quality in care could not be measured without addressing concerns such as health care providers caring attitudes or respect for patient preferences and informational needs [95, 115, 116].

### Existing patient-reported experience measures (Results for Objective 2)

For objective two, we included 128 [17–22, 24, 25, 43–46, 48, 50–91, 119–160, 160–191] (Supplementary material 7) reports that described 113 unique PREMs (Supplementary materials 4 and 6). However, 83 (73%) PREMs were excluded during data extraction as they referred to other aspects of care quality than transitions between healthcare settings or otherwise deviated from our specified phenomenon (Supplementary material 6). The Consumer Assessment of Healthcare Providers and Systems (CAHPS) [192–195] questionnaires were most frequently referred to, [17, 18, 20, 22, 81, 119, 121, 123] but we did not find the items relevant according to the construct definition in objective 1. The two relevant PREMs that were most frequently referred to were Nijmegen Continuity Questionnaire [19, 24, 68–72, 172] and Patient Assessment of Chronic Illness Care (PACIC) [17, 22, 24, 75–77, 79, 196]. 

In the data extraction process, we identified different ways of formulating items. Some items were worded from a medical system perspective on quality i.e. “My physical pain was controlled as well as possible”, [197] whereas others were articulated from a patient-centred perspective i.e. “My treatment fits my needs” [43]. Yet, other items were specific to a certain contextual system infrastructure i.e. “The specialist makes out the first prescription for the treatment he/she prescribes me”, [44] or they were disease-specific [61]. However, we did identify PREMs with a more generic wording such as, “Were there times when you had to repeat information that should have been in your care records?” [198].

### Adequate reflection of patients’ experience (results for objective 3)

We extracted 30 PREMs that pertained to patient-experience of quality of transitions in healthcare settings. To assess the relevance and comprehensiveness of the identified PREMs, the items of each PREM were plotted according to the 12 subthemes identified for objective 1 (Table 3). Twenty-two PREMs had items related to at least five of the 12 subthemes (Table 3). To focus our review on the most comprehensive PREMs, we critically appraised the selected 22 PREMs. The Alberta Continuity of Services Scale – Mental Health (ACSS-MH), [43] the Person-Centered Coordinated Care Experience Questionnaire (P3CEQ), [74] and the Patient Experience of Integrated Care Scale (PEICS) [84] had adequate content validity, however, they were disease-specific and/or did not have items in all themes identified in objective 1 (Table 3). The remaining 17 PREMs had doubtful or inadequate content validity. Despite P3CEQ and PEIC having been adequately tested for content validity we do not find them comprehensive according to our conceptualization of the construct (Objective 1) and thus not content valid.

## Discussion

The overall aim of this scoping review was to define the concept of patient-experienced quality in healthcare transitions and map existing content valid PREMs relevant for measuring this concept. We found the construct of patient-reported experience of transitions in healthcare to consist of two domains – system/organization and human-relation. However, in summarizing the 27 qualitative reports for this review, we found some inconsistency and lack of clarity in the conceptualization and understanding of patient-experienced quality in healthcare transitions. Although some reports identified the construct of patient-experienced quality in healthcare transitions to consist of two main domains (organization and human-relational), [19, 20, 62, 102, 108, 115] others disagreed [95, 111] and leaned towards the Institute of Medicine’s framework for quality with five or more domains [36]. The two-dimensional model is, however, supported by both qualitative conceptualization [102, 108, 115] and testing of measurement properties, [19, 20, 62] whereas the Institute of Medicine’s framework is not. In addition to inconsistency in domains, there was a general inconsistency in the number and terminology for themes [94, 111] and formulation of items concerning patient-centeredness. [43, 197] As items in PREMs may be approached by respondents much the same as a dialogue, [199] we believe a person-centred approach to item formulation may provide the best opportunity for patients to assess quality of care appropriately. Several reports suggest further research into the conceptualization and understanding of patient-experience with care transitions [94, 98, 110, 118]. Due to the variations in how the construct is defined, assessing the content validity of current PREMs becomes challenging [27, 28]. Therefore, we support the suggestion of further research into the conceptualization of patient-experienced quality in healthcare transitions.

We identified 30 PREMs that reflected at least one relevant aspect of the construct but none that were comprehensive reflections of generic patient populations’ experiences of transitions in healthcare settings. This finding aligns with the conclusion of the included reviews of instruments [17–22, 24, 25, 62, 177]. It is surprising that we have not identified a content-valid PREM given the large number of reports (128) and unique PREMs (113) included. This may be associated with the lack of clarity in the construct of patient-experienced quality in healthcare transitions. In the future, a generic PREM should be developed to make cross-comparison between studies and healthcare organizations possible. A collective effort to test and use a generic PREM might also support further development and/or understanding of the construct. This, however, would entail a generic approach to item formulation, as seen in P3CEQ, [198] rather than a context-specific approach [44].

It may be a limitation in our study that our search was imprecise with the inclusion of patient satisfaction in the search terms. However, the sensitivity of our search originates from inclusion of patient satisfaction in the search terms, and we consider the strength of this sensitivity to out way the imprecision by securing a comprehensive review. The comprehensiveness of our search resulted in a large number of records to be reviewed, and thus many reviewers to accommodate for time constraints. We attempted to compensate for a potentially low inter-rater reliability with calibration meetings. Despite of this, the number of reviewers may have been a limitation to the inclusion of all relevant, and only relevant reports. Furthermore, our pragmatic decision of excluding original qualitative studies from this review could have been a limitation to the comprehensiveness of our results. Incorporating the 24 qualitative reports has sufficiently advanced our comprehension of the existing literature to address objective 1. This is corroborated by the absence of new themes identified during data extraction from recent reports. The data extraction performed primarily by one reviewer may have caused some imprecision. However, as we have identified more PREMs than the included reviews of instruments, [17–22, 24, 25, 62, 177] this does not seem to be the case. While our scoping review does exhibit certain limitations, the thoroughness of our search and the inclusive methodology employed in comprehending and evaluating patient-experienced quality during healthcare transitions have nonetheless generated novel and significant insights.

CTM [56, 200] and PACIC [196] are widely used measures of patient-experience of transitions in healthcare settings and Nijmegen Continuity Questionnaire [70, 172] is often used for measurement of continuity. However, we found P3CEQ [73, 74], and PEICS [84] to have more adequately tested content validity for generic measurement of patient-experienced quality in healthcare transitions. With seventeen [73, 84] respectively twenty items for the questionnaires, we do not expect any one of them to be more challenging for participants to respond to. P3CEQ had been found to be difficult to use in an older population [164], but this is likely to be true true for both questionnaires. The questionnaires have some overlap in themes and some differences. As P3CEQ has been more thoroughly tested using item response theory methods [73, 74, 191] we recommend the use of P3CEQ if the questionnaire has face validity for the intended purpose. We do still find though, that some items relating to kindness in care [95] are missing and that neither P3CEQ nor PEICS are comprehensive measures of patient-experienced quality in healthcare transitions according to the conceptualizations we have identified.

In light of our findings, it is plausible that items extracted from the most relevant and comprehensive PREMs, with a focus on generic formulations for infrastructure and disease, would reflect the construct of patient-experienced quality in healthcare transitions adequately. Furthermore, as there is some consensus that quality healthcare transitions occur when organisational structures are flexible and sensitive to patient preferences, [96, 101–104, 107, 114] it seems advantageous to apply this knowledge in item extraction and/or formulation. As described, the construct seems to be unclear or imprecisely defined. Therefore, a process of extracting and/or formulating items should be undertaken systematically and iteratively with patient involvement and openness to re-evaluating the definition of the construct. A PREM revised by the outlined approach may support valid and reliable evaluation of the effectiveness, safety, and efficiency of healthcare provision.

With this scoping review we share an overview of available PREMs for assessment of patient- experienced quality of healthcare in pathways with transitions between settings. Our review may have implications for assessment of transitional care in the future, as we do not recommend continued use of CTM [56, 200]. Comprehensive and valid measurement of patients’ experiences is pivotal to securing high quality, safe healthcare for patients with complex disease [1, 201] and we would welcome a collaborative, international effort to define the construct and futher assess the existing PREMs or co-create a measure on the basis of the existing PREMs.

## Conclusion

In the literature, we identified several conceptual models that referred to aspects of patients’ experience with the quality of healthcare transitions. We consider a model with two domains likely to be adequate, however, a more comprehensive analysis and adequate definition of the construct is needed.

Thirty PREMs assessing at least one aspect of patients’ experience of transitions in healthcare were identified. However, we did not consider any of the PREMs to be content valid to measure patient-experienced quality in healthcare transitions generically according to the conceptual models we identified. It is possible that items extracted from the identified questionnaires can be combined for a content-valid PREM. We call for further exploration into the construct of patient experience with healthcare transitions and testing of models to produce a content-valid PREM suitable for generic assessment of patients’ experiences with the quality of healthcare transitions.

### Supplementary Information

Supplementary Material 1.

Supplementary Material 2.

Supplementary Material 3.

Supplementary Material 4.

Supplementary Material 5.

Supplementary Material 6.

Supplementary Material 7.

## Data Availability

The datasets used and/or analysed during the current study are available from the corresponding author on reasonable request.
