# Supplementary material for: Content validity of patient-reported measures evaluating experiences of the quality of transitions in healthcare settings—a scoping review
Source: BMC Health Serv Res. 2024 Jul 22;24:828. doi: 10.1186/s12913-024-11298-0 (PMC11265152; doi:10.1186/s12913-024-11298-0)
Supplement: Supplementary file 1 — Supplementary Material 1. [file 12913_2024_11298_MOESM1_ESM.docx]

The initial scoping search was inspired by a 2016 Danish report on development of a PREM on quality in cross-sectoral care (21). We began our scoping search and the development of our search strategy in Ovid Medline by searching for Mesh terms on September 20th, 2021. The search strategy was developed by first author SW in collaboration with a research librarian and reviewed by co-author SMH who had prior knowledge of systematic literature review. Based on expert knowledge in our research group, we decided to search for literature from the year 2000 and onwards, as the focus on integrated care seemed to have emerged around late 1990 and early 2000. Our research aims warranted a broad variety of literature, founded in different research traditions. Therefore, we chose to search Medline Ovid, Embase Ovid and Cinahl. We wanted to narrow our search to published, peer-reviewed literature and decided to limit our search to these three databases.

## Combination

We combined the “Setting” and the “Phenomenon of interest” search using AND. The combination and selection of search terms was an iterative process headed by primary researcher SW, supported by a librarian and co-authors SMH, LM, and HHL. The search strategy was run and evaluated on 4 separate occasions by:

- Screening the first 100 hits for relevance. Based on this screening, it was decided to remove the search terms (person* adj centr*), (people* adj centr*), and (patient* adj centr*) from the *setting* block. These terms seemed to be unprecise in defining the setting. Furthermore, person-centred care is a sub-category of integrated care, which is contained in the search.
- Screening the first 100 hits for additional search terms in title, abstract and key words.
- Discussion of the search terms in the author group.
- Screening the first 100 hits for key articles, that SW was familiar with. This led to the addition of (coordinat* adjn care*) in the *settings* block.

The adj Boolean operator was evaluated with different numbers to screen how it affected precision and specificity.

# Search

The search syntaxes were planned to be run on November 25^th^ 2021, but re-scheduled to December 7^th^ 2021 and re-run on May 27^th^ 2024:

|  | **MEDLINE Ovid (Ovid MEDLINE(R) ALL)** |
| --- | --- |
| 1 | "continuity of patient care"/ or patient discharge/ or patient handoff/ or patient transfer/ or retention in care/ or transitional care/ |
| 2 | *"Delivery of Health Care, Integrated"/ |
| 3 | (care adj2 continu*).ab,kf,ti. |
| 4 | (care adj2 across adj5 sectors).ab,kf,ti. |
| 5 | (care adj2 ?cross adj5 sector*).ab,kf,ti. |
| 6 | (inter* adj2 sector* adj2 care).ab,kf,ti. |
| 7 | (integrat* adj care).ab,kf,ti. |
| 8 | (transition* adj2 care).ab,kf,ti. |
| 9 | (coordinat* adj3 care).ab,kf,ti. |
| 10 | 1 or 2 or 3 or 4 or 5 or 6 or 7 or 8 or 9 |
| 11 | exp Patient Satisfaction/ |
| 12 | (patient* adj1 experience*).ab,ti. |
| 13 | (patient* adj1 perspective*).ab,ti. |
| 14 | (patient* adj2 view*).ab,kf,ti. |
| 15 | (patient* adj2 attitude*).ab,kf,ti. |
| 16 | (patient* adj2 satisf*).ab,kf,ti. |
| 17 | (patient* adj2 involvement*).ab,kf,ti. |
| 18 | (user* adj2 perspective*).ab,kf,ti. |
| 19 | (user* adj2 view*).ab,kf,ti. |
| 20 | (user* adj2 involvement*).ab,kf,ti. |
| 21 | (user* adj2 attitude*).ab,kf,ti. |
| 22 | (user* adj2 satisf*).ab,kf,ti. |
| 23 | (user* adj2 involvement*).ab,kf,ti. |
| 24 | (people* adj1 experience*).ab,ti. |
| 25 | (people* adj1 perspective*).ab,ti. |
| 26 | (people* adj2 view*).ab,kf,ti. |
| 27 | (people* adj2 attitude*).ab,kf,ti. |
| 28 | (people* adj2 satisf*).ab,kf,ti. |
| 29 | (people* adj2 involvement*).ab,kf,ti. |
| 30 | 11 or 12 or 13 or 14 or 15 or 16 or 17 or 18 or 19 or 20 or 21 or 22 or 23 or 24 or 25 or 26 or 27 or 28 or 29 |
| 31 | 10 and 30 |
| 32 | Limit 31 to yr=”2000-Current” |
| 33 | Limit 32 to yr=”all child (0 to 18 years)” |
| 34 | Limit 33 to yr=”all adult (19 plus years)” |
| 35 | 33 not 34 |
| 36 | 32 not 35 |
|  | **EMBASE Ovid** |
| 1 | exp integrated health care system/ |
| 2 | exp transitional care/ |
| 3 | (care adj2 continu*).ab,kf,ti. |
| 4 | (care adj2 across adj5 sectors).ab,kf,ti. |
| 5 | (care adj2 ?cross adj5 sector*).ab,kf,ti. |
| 6 | (inter* adj2 sector* adj2 care).ab,kf,ti. |
| 7 | (integrat* adj2 care).ab,kf,ti. |
| 8 | (transition* adj2 care).ab,kf,ti. |
| 9 | (coordinat* adj3 care).ab,kf,ti. |
| 10 | 1 or 2 or 3 or 4 or 5 or 6 or 7 or 8 or 9 |
| 11 | *patient satisfaction/ or *patient attitude/ |
| 12 | (patient* adj1 experience*).ab,ti. |
| 13 | (patient* adj1 perspective*).ab,ti. |
| 14 | (patient* adj2 view*).ab,kf,ti. |
| 15 | (patient* adj2 attitude*).ab,kf,ti. |
| 16 | (patient* adj2 satisf*).ab,kf,ti. |
| 17 | (patient* adj2 involvement*).ab,kf,ti. |
| 18 | (user* adj2 perspective*).ab,kf,ti. |
| 19 | (user* adj2 view*).ab,kf,ti. |
| 20 | (user* adj2 involvement*).ab,kf,ti. |
| 21 | (user* adj2 attitude*).ab,kf,ti. |
| 22 | (user* adj2 satisf*).ab,kf,ti. |
| 23 | (user* adj2 involvement*).ab,kf,ti. |
| 24 | (people* adj1 experience*).ab,ti. |
| 25 | (people* adj1 perspective*).ab,ti. |
| 26 | (people* adj2 view*).ab,kf,ti. |
| 27 | (people* adj2 attitude*).ab,kf,ti. |
| 28 | (people* adj2 satisf*).ab,kf,ti. |
| 29 | (people* adj2 involvement*).ab,kf,ti. |
| 30 | 11 or 12 or 13 or 14 or 15 or 16 or 17 or 18 or 19 or 20 or 21 or 22 or 23 or 24 or 25 or 26 or 27 or 28 or 29 |
| 31 | 10 and 30 |
| 32 | Limit 31 to child <unspecified age> |
| 33 | Limit 32 to adult <18 to 64 years> |
| 34 | Limit 33 to aged <65+ years> |
| 35 | 32 not 33 not 34 |
| 36 | 31 not 35 |
| 37 | Limit 36 to yr=”2000-current” |
|  | **EBSCOhost CINAHL** |
| 1 | (MH "Continuity of Patient Care+") |
| 2 | (MM "Transitional Care") |
| 3 | TI(integrat* N3 care*) OR AB(integrate* N3 care*) |
| 4 | TI(transition* N2 care*) OR AB(transition* N2 care*) |
| 5 | TI(care N1 across N5 sector*) OR AB(care N1 across N5 sector*) |
| 6 | TI(care N2 ?cross N5 sector*) OR AB(care N2 ?cross N5 sector*) |
| 7 | TI(inter* N2 sector* N2 care) OR AB(inter* N2 sector* N2 care) |
| 8 | TI(care N2 continu*) OR AB(care N2 continu*) |
| 9 | TI(coordinat* N3 care* ) OR AB(coordinat* N3 care* ) |
| 10 | 1 or 2 or 3 or 4 or 5 or 6 or 7 or 8 or 9 |
| 11 | (MH "Patient Satisfaction+") |
| 12 | (MM "Patient Attitudes") |
| 13 | TI(patient* N1 experience*) OR AB(patient* N1 experience*) |
| 14 | TI(patient* N1 perspective*) OR AB(patient* N1 perspective*) |
| 15 | TI(patient* N1 view*) OR AB(patient* N1 view*) |
| 16 | TI(patient* N2 satisf*) OR AB(patient* N2 satisf*) |
| 17 | TI(patient* N2 attitude*) OR AB(patient* N2 attitude*) |
| 18 | TI(patient* N2 involvement*) OR AB(patient* N2 involvement*) |
| 19 | TI(user* N2 experience*) OR AB(user* N2 experience*) |
| 20 | TI(user* N2 perspective*) OR AB(user* N2 perspective*) |
| 21 | TI(user* N2 view*) OR AB(user* N2 view*) |
| 22 | TI(user* N2 involvement*) OR AB(user* N2 involvement*) |
| 23 | TI(user* N2 satisf*) OR AB(user* N2 satisf*) |
| 24 | TI(user* N2 attitude*) OR AB(user* N2 attitude*) |
| 25 | TI(people* N1 experience*) OR AB(people* N1 experience*) |
| 26 | TI(people* N1 perspective*) OR AB(people* N1 perspective*) |
| 27 | TI(people* N1 view*) OR AB(people* N1 view*) |
| 28 | TI(people* N2 satisf*) OR AB(people* N2 satisf*) |
| 29 | TI(people* N2 attitude*) OR AB(people* N2 attitude*) |
| 30 | TI(people* N2 involvement*) OR AB(people* N2 involvement*) |
| 31 | 11 or 12 or 13 or 14 or 15 or 16 or 17 or 18 or 19 or 20 or 21 or 22 or 23 or 24 or 25 or 26 or 27 or 28 or 29 or 30 |
| 32 | 10 and 31 |
| 33 | Limiters: Published Date 20000101- |
