# Supplementary material for: Content validity of patient-reported measures evaluating experiences of the quality of transitions in healthcare settings—a scoping review
Source: BMC Health Serv Res. 2024 Jul 22;24:828. doi: 10.1186/s12913-024-11298-0 (PMC11265152; doi:10.1186/s12913-024-11298-0)
Supplement: Supplementary file 2 — Supplementary Material 2. [file 12913_2024_11298_MOESM2_ESM.docx]

### Inclusion

- RCTs as well as qualitative, and quantitative observational studies were included. Reviews were also included.
- The patient perspective of WHAT patient experienced quality or satisfaction in cross sectoral care covers was pivotal. If it was merely reported that “patients were more satisfied”, the concept was not enlightened thoroughly enough.
- All studies reporting the development, reliability testing, or validation of a measurement that could capture transitions in healthcare settings were included. Also translation studies.

### Exclusion

- For studies reporting on discharge follow-up, it could be difficult to define whether they were single-setting or cross-sectoral. If the only actor was the hospital, and GP or municipality were not involved in the intervention or evaluation, and if patients only had contact with the hospital during follow-up care, the studies were excluded.
- Several studies reported on integrated care clinics. This could be clinics that integrated GP-care and behavioral care, or clinics that integrated medical professionals, dieticians, physiotherapists, diabetes care and/or other setups. We chose to view these integrated clinics as single-settings (primary sector) rather than cross-sectoral collaborations, and studies reporting on these clinics were excluded.
