# Supplementary material for: Content validity of patient-reported measures evaluating experiences of the quality of transitions in healthcare settings—a scoping review
Source: BMC Health Serv Res. 2024 Jul 22;24:828. doi: 10.1186/s12913-024-11298-0 (PMC11265152; doi:10.1186/s12913-024-11298-0)
Supplement: Supplementary file 3 — Supplementary Material 3. [file 12913_2024_11298_MOESM3_ESM.docx]

| Title | User Experience and Care Integration in Transitional Care for Older People From Hospital to Home: A Meta-Synthesis | The transition of palliative care from the hospital to the home: a narrative review of experiences of patients and family caretakers | Patient and caregiver experiences on care transitions for adults with a hip fracture: a scoping review | Do the Institute of Medicine’s (IOM’s) dimensions of quality capture the current meaning of quality in health care? – An integrative review | Older multimorbid patients’ experiences on integration of services: a systematic review | Hospital Palliative Care Teams and Post-Acute Care in Nursing Facilities |
| --- | --- | --- | --- | --- | --- | --- |
| Author(s) | Jacqueline Allen, Alison M. Hutchinson, Rhonda Brown, Patricia M. Livingston | Mauricio Arias Rojas, Cristina García-Vivar | Maliha Asifa, Lauren Cadela, Kerry Kuluskib, Amanda C. Everalla, Sara J. T. Guilchera | Michelle Beattie, Ashley Shepherd, Brian Howieson | Lilian Keene Boye, Christian Backer Mogensen, Tine Mechlenborg, Frans Boch Waldorff, Pernille Tanggaard Andersen | Joan G. Carpenter |
| Year of publication | 2016 | 2015 | 2020 | 2012 | 2019 | 2017 |
| Countries of origin |  |  | Canada (n 6), Norway (n 1), the United States (n 1), Denmark (n 1), and Australia (n 1). Multiple countries: Czech Republic, France, Germany, Poland, Portugal, Spain and Turkey. | Canada (1) England (5), Ireland (1), Netherlands (2), Scotland (1), USA (12) | Australia, Canada (2), Denmark, Norway, Sweden, United Kingdom (2), USA, | Australia, Germany, Norway, United Kingdom, United States (8) |
| Setting | Hospital and community based providers | Palliative care attention team, hospices or palliative care unit. | Transition from hospital – orthopaedic surgery. | Primary care, hospital, general medicine, disabilities service. | Not reported | Hospital to nursing care facility - Palliative care |
| Aim(s) | To improve understanding about user experience and care integration during the discharge and transitional care of older people with multiple chronic health problems. | To identify, analyze, and synthesize the qualitative studies published on the experiences of patients and family caretakers during the transition of palliative care from the hospital to the home. | To determine what is known about the perspectives of patients and caregivers on care transitions for adults recovering from a hip fracture. | To determine whether the IOM dimensions (IOM, 2001) capture the current meaning of quality in health care. | To synthesize the manner in which older patients with multi-morbidity experience the level of integration of health care services and to identify barriers to continuity of care | To present the findings of an integrative literature review that focuses on the care of patients discharged to nursing facilities following a hospital-based palliative care consult. |
| Review design | Meta-synthesis | Narrative review | Scoping review | Integrative review | Systematic review | Integrative review |
| Theoretical framework and/or additional key features | The synthesis was a conceptualization of themes derived in the translation phase. It was conducted by comparing and contrasting (1) user experiences of older people and carers with those of health providers, and (2) user experiences of hospital- with community-based providers. | Not reported | Arksey and O’Malley’s methodological framework with updates by Levac et al. | The IOM’s six dimensions of quality were used to extract the themes from the literature. Additional data was also extracted and integrated into themes. | Narrative presentation. Thematic synthesis. | Iterative approach to analysis with constant comparison techniques to explore patterns and themes. |
| Use of the PRISMA guideline | Not reported | Not reported | Yes (for Scoping reviews) | Not reported | Yes | Yes |
| Electronic resources | CINAHL, Psychinfo, Medline,  Proquest, Academic Search Complete, Masterfile Premier, SocIndex, Humanities, Social Sciences Collection, Cochrane Collaboration, Joanna Briggs Institute, Google Scholar | PubMed, Cochrane Central, ScienceDirect, Ovid Nursing, CINALH, Scielo, Bireme | MEDLINE, EMBASE, PsycINFO, Allied and Complementary Medicine, Cumulative Index to Nursing and Allied Health Literature, Applied Social Sciences Index and Abstracts and Cochrane Database of Systematic Reviews. Grey literature: TSpace, Health Quality Ontario, Bone and Joint Canada, Canadian Institute for Health Information and the World Health Organization websites. | MEDLINE, CINAHL | Scopus, Embase, CINAHL, MEDLINE. | PubMED, CINAHL, Ageline, PSYCINFO |
| Search strategies | Concept group 1: ‘discharge planning,’ ‘hospital discharge,’ ‘discharge care pathways,’ ‘discharge care protocols,’ ‘transitional care,’ ‘transitional care pathways,’ ‘transitional care protocols’ Added to Concept Group 2: “from the inpatient setting to the home” Added to Concept Group 3: user experience/patient experience/aged care/aging/ geriatrics/gerontology/older person care, and community/ home care/primary care/ domiciliary care | (Family OR Caregiver OR Patient) AND (Perspective OR Perception OR Experience) AND ( Terminal Care OR Palliative Care OR end of life care) AND ( Patient discharge OR Discharge planning OR hospital discharge) | Search terms included: hip fracture, transitional care, care continuum, patient transfer, care transitions | *Quality of Health Care/or *Quality Assurance, Health Care/or *Quality Indicators, Health Care/ | Search terms included Comorbidity, Multimorbidity, or frailty AND aged, elderly, or senior AND Patient Satisfaction, preference, attitude, perception, or experience AND Continuity of Patient Care, integrated care, inter sectoral collaboration, cooperation, or continuity of care | "palliative care AND hospital AND discharge [all] AND (nursing home OR nursing facility OR extanded care facility)" |
| Year coverage | January 1990 - August 2014 | January 2000 - February 2014 | January 1, 2000 - July 3, 2018 | 2000 - 2010 | Not reported | Inception to February 2016. |
| Languages | English | English and Spanish | Not reported |  | English, Danish, Swedish or Norwegian | English |
| Quality appraisal | Qualitative Research Checklist | Not reported | Not reported | Graded according to the hierarchy of evidence - National Institute for Health and Clinical Excellence (NICE) Framework | CoreQ. | None reported |
| Population | Older people with multi-morbidity, their carers, and community- or hospital-based health providers. | People in need of palliative care and their caretakers. | Patients with hip fracture and caregivers. | Not reported | People over the age of 65 with multi-morbidity | Clinicians, adult patients, and caregivers. |
| Inclusion criteria | (1) report, in a peer-reviewed journal, primary data about the user experience in transitional care; (2) use a qualitative design or mixed methods design with a qualitative component that included analysis resulting in key concepts, metaphors, and themes; (3) be published in English; (4) focus on multiple chronic conditions rather than a specific disease; (5) focus on appraising the user experience from the perspectives of older people, their carers, and community- or hospital-based health providers | Articles in full text, with qualitative or mixed focus and which will approach the experiences of PC adult patients with any underlying disease on hospital discharge, family caretakers and/or relatives on the transition between hospital and home contexts | (1) included a study sample of patients with hip fracture over the age of 18; (2) primarily focused on patient and caregiver experiences; (3) addressed the topic of transitions in care; (4) were peer-reviewed or in the grey literature; and (5) published between January 1, 2000 to July 3, 2018. | Papers were included if the main focus of the paper was quality in relation to health care as defined or utilised by the authors or study participants of the papers. The population was specifically in relation to patient, service user or any other term used to describe those accessing or providing health care. | The studies had to explore the patients’ viewpoints and address aspects such as their experience, and opinions about transitions, their relationship with their health care providers and relatives, the transition of information, and consistency of care and personnel and the health care system they were navigating within. The study participants had to be patients aged 65 years or older. The study participants had to be patients with multimorbidity. | To be included, a study needed to address inpatient hospital palliative care, illness trajectory after hospital discharge, and/or post hospitalization discharge follow-up involving the nursing facility setting. |
| Exclusion criteria | Quantitative, discharge only, not older people, not user experience, re-admission only, single disease, secondary analysis | Not reported | (1) books, book chapters, opinion pieces, editorials, study protocols, case laws, trial papers; (2) studies performed on non-humans; and (3) conference and poster abstracts without a full-text article | Performance management/improvement, quality improvement methodology, service re-design, clinical pathways or indicators, Risks/interventions specific to disease/illness/procedure/ diagnosis or prognosis, Irrelevant papers not defining quality of health care, Animal, in-vitro or laboratory, Eastern health care – namely Asia, India sub continental, Far East, Middle East, Near East, Dentistry, Nursing home or residential care, End of life or terminal care, Social care. | not written in English, Danish, Norwegian, or Swedish. Not peer-reviewed, published articles. | Additional studies where excluded if authors reported only on tool testing and development, hospital mortality or in-hospital outcomes, hospice outcomes palliative home care, or home discharge without inclusion of a nursing facility. Studies that focused on hospice care where excluded due to the empirical evidence that palliative care outcomes are improved when nursing home residents recieve hospice care. |
| Number of studies identified in search | 462 | 12452 | 1107 | 171 | 1693 | 53 |
| Number of studies included | 20 | 14 | 11 | 22 | 9 | 12 |
| List of included studies by author and publication year | Armitage & Kavanagh, 1995, 1996; Bull, 1992, 1994; Bull & Roberts, 2001; Byrne et al., 2011; Chapin et al., 2014; Coleman et al., 2002; Foust et al., 2012; Graham et al., 2009; Grimmer et al., 2004; Huby et al., 2007; LeClerc et al., 2002; McKeown, 2007, McWilliam, 1992; McWilliam & Sangster, 1994; Procter et al., 2001; Rydeman & Törnkvist, 2006, 2010; Zakrajsek et al., 2013) | Hasson 2010, Murray 2002, Rabow 2004, Benza 2011, McIlfatrick 2007, Hayle 2013, Hanratty 2012, Wennman-Larsen 2002, Holley 2009, Moore 2013, Grimmer 2000, Strachan 2009, Tallman 2012, Stajduhar 2013. | Elliot 2009, Giosa 2014, Glenny 2013, Groene 2015, Jensen 2017, Killington 2016, Nahm 2010, Schiller 2015, Storm 2014, Toscan 2013. | Attree 2001, Baker 2001, Barels 2009a, Barels 2009b, Bassett 2010, Brook 2000, Campbell 2000, Chilgren 2008, English 2002, Frist 2000, Haggerty 2007, Heenan 2010, Hickman 2001, Howie 2004, Jones 2010, Larrabee 2001, Manning 2006, O´Reilly 2007, Russell 2007, Sipkoff 2014, Sofaer 2005, Williams 2000. | Andreasen 2015, Arendts 2015, Bayliss 2008, Butterworth 2014, Foss 2011, Gabrielson-Järhult 2016, Gill 2014, Neiteman 2015, Sheaff 2017. | Baldwin 2013, Benzar 2011, Blackford 2001, Brody 2010, Catic 2013, Enguidanos 2012, Fromme 2006, Gade 2008, Gerrard 2011, Kötzsch 2014, Tallman 2012, Thon 2013. |
| Qualitative themes | Care integration practices and absence of care integration. Four themes: (I) ‘Who is taking care of what? Trying to work together’; (II) ‘Falling short of the mark’; (III) ‘A proper discharge’; (IV) ‘You adjust somehow.’ | Six principal themes: (I) The dyad and its knowledge regarding the diagnosis and prognosis; (II) Emotions experienced by the family caretaker and patient during discharge; (III) Effective communication among those involved in caring; (IV) Education for caring for the person at home; (VI) Continuous support to the dyad at home, and care overload. | Two main themes: (I) Challenges affecting care transitions: Lack of information sharing, Role confusion, disorganized discharge planning. (II) Suggestions for improving care transitions: Increasing written information, Offering a patient representative role, using technology for facilitating communication, increasing geriatrician involvement. | Two additional dimensions of quality; caring and navigating the health care system. All IOM dimensions were prevalent in the literature, but not necessarily sufficient to capture the wholeness of quality in current health care. | Three main themes: (I) Involved in decision-making; (II) Successful integration and sense of security during transfer; (III) Relationship to healthcare provider. | Four themes: (I) Symptom management; (II) Communication; (III) Care continuity; (IV) Patient survival. |

| Title | Patient experiences of integrated care within the United Kingdom: A systematic review | The role of hospitals in bridging the care continuum: a systematic review of coordination of care and follow-up for adults with chronic conditions. | The relationship between integrated care and cancer patient experience: A scoping review of the evidence. | Older persons' experiences of adapting to daily life at home after hospital discharge: a qualitative metasummary. | Patient perspectives on primary care and oncology care coordination in the context of multiple chronic conditions: A systematic review | What does integrated care mean from an older person’s perspective? A scoping review |
| --- | --- | --- | --- | --- | --- | --- |
| Author(s) | Lea Davidson, Jason Scott, Natalie Forster | Melissa De Regge, Kaat De Pourcq, Bert Meijboom, Jeroen Trybou, Eric Mortier, Kristof Eeckloo. | Silvia Foglino, Franscesca Bravi, Elisa Carretta, Maria Pia Fantini, Mark J Dobrow, Adalsteinn D Brown. | Christine Hillestad Hestevik, Marianne Molin, Jonas Debesay, Astrid Bergland, Asta Bye. | Natalie S. Hohmann, Cassidi C. McDaniel, S. Walker Mason, Winson Y. Cheung, Michelle S. Williams, Carolina Salvador, Edith K. Graves, Christina N. Camp, Chiahung Chou | Michael T. Lawless, Amy Marshall, Manasi Murthy Mittinty, Gillian Harvey |
| Year of publication | 2021 | 2017 | 2016 | 2019 | 2020 | 2020 |
| Countries of origin | England (11), Scotland (3), Wales (1), UK (1 - not named sites) | Africa, Asia (3), Australia (2), Canada (2), Ireland, Italy, Netherlands (3), Spain (3), Sweden, United Kingdom (5), United States (10) | 23 from USA, Canada, Australia, New Zealand, UK. 5 from Denmark, Spain, Sweden. 2 from Korea, Japan. | Australia (3), Canada, Denmark, Ireland, New Zealand (2), Sweden (2), United Kingdom, USA (2) | Australia (4), Canada (4), England, Netherlands, USA (12), | Australia (3), Canada (5), Denmark, Netherlands (3), Sweden (2), United Kingdom (9), USA (6). 1 international survey including 11 countries. |
| Setting | United Kingdom. Integrated care models with a change in coordination or integration of service delivery. | Hospitals with coordinating roles. | Cancer services. Large healthcare centres like teaching hospitals. | At home after hospital discharge. | Primary care and oncology care continuum | No limitations on health care setting or geographical setting. |
| Aim/purpose | To develop an understanding of the current integrated care models in the UK and develop an understanding of current integrated care models in the UK and determine whether evaluations of patient experience reflect a person-centered coordinated care approach. | To examine current evidence and provide a structured, comprehensive overview of the role of hospitals in the downstream coordination and follow-up care of chronically ill patients. | To describe the extent, scope and findings of the existing literature on the relationship between the integration of cancer services and patient experience. | To integrate current international findings in order to enhance the understanding of older persons’ experiences of adapting to daily life at home after hospital discharge. | What are the perceptions of patients with both cancer and MCC regarding best practices for care coordination between PCPs and oncologists during the cancer care continuum? | 1. How do older patients define their views and experiences of integrated care? 2. What are the barriers and enablers of quality integrated care from an older person’s perspective? 3. What is the quality of the literature on older patients’ perspectives on integrated care? 4. What are the potential implications for the design and implementation of integrated care programmes for older people? |
| Study design | Systematic review | Systematic review. | Scoping review | Qualitative meta-summary | Systematic review | Scoping review |
| Theoretical framework and/or additional key features | PRISMA. Framework synthesis. The Rainbow model of Integrated Care was used to code the processes of integration data within the studies. The Measuring Integrated Care Framework was selected to code data relating to patient experience. | Not stated clearly | Scoping review guided by Levac et al. Qualitative conventional content analysis. | Methodological framework of Sandelowski and Barroso. Qualitative meta-summary as a quantitatively oriented aggregation approach to research synthesis. | Grounded theory for evaluating data and developing framework. PRISMA | The scoping review methodology outlined by Arksey and O’Malley. PRISMA-ScR. A narrative descriptive technique for synthesis of findings. |
| Search databases | ProQuest, EBSCO, Cochrane Library | Embase, Pubmed, Cinahl, EBSCO, Web of Sciences, The Cochrane Library. | PubMed, Embase, Scopus. | Medline, Embase, Academic Search Premier, Cinahl, PsycINFO. | PubMed, CINAHL, PsycINFO | EMBASE, CINAHL, PubMed, ProQuest, Open Grey, Google Scholar |
| Key search terms | (experience* OR satisfaction OR perc* OR value* OR perspective* OR view* OR feedback (ABSTRACT)) AND (patient* OR client* OR user* OR consumer* OR carer* OR men OR women (ABSTRACT)) AND (integrat* OR coordinat* OR co-ordinat* OR collabor* OR continui* OR joint OR multidisciplinary OR partner* OR "single point of access" (ABSTRACT)) AND health OR social OR system* OR care OR team* OR service* OR housing (ABSTRACT)) AND ("United Kingdom" OR "UK" OR England OR Scotland OR Wales OR "Northern Ireland" OR "Great Brittain" (ANYWHERE)) | ("delivery of health care, integrated" OR "transmural care" OR "chain care" OR "chain of care" OR "care chain" OR "care continuity, continuum of care" OR "case management" OR "disease management" OR "health network" OR "care network" Or "patient care management" OR "long term care" OR "transitional care" OR "discharge care" OR "hospital discharge" OR "coordination of care" OR "care coordination") AND (hospitals OR "inpatient care" OR "inpatient setting" OR "hospitalization") AND ("chronic disease" OR "chronic illness" OR "chronocally ill" OR "chronic condition" OR comorbidity OR multimorbidity OR "multiple chronic conditions") | PICO; P: cancer patient (also replacing the word cancer" for its synonyms); I: continuity of care, coordination of care, integration of care, patient-centered care, case management; O: patient perspective, patient experience, aptient satisfaction. | Aged, older patient, frail, elderly linked with patient discharge, patient transfer, patient handover, transitional care, hospital to home, hospital to municipal, hospital to community, patient (satisfaction, perception, experience, perspective, view) and interview or focus groups | Search terms included variations of “cancer,” “comorbidities,” “care coordination,” and “patient perception.” | (‘Aged’[mh] OR older patient*[tw] OR elderly patient*[tw]) AND (views*[tw] OR perspective*[tw] OR expectation*[tw] OR experience*[tw]) AND (‘Delivery of health care, integrated’[mh] OR integrated care[tw] OR fragmented care[tw] OR continued care[tw] OR preventative care service*[tw] OR preventive services*[tw] OR curative service*[tw]) |
| Search dates/years | None reported | 1st January 1995 - 28th February 2016 | None reported | 2006 - 2017 | 1 January 2008 - 19 September 2018 | June 2008 - July 2019 |
| Languages | English | English | English and italian | English | English | English |
| Quality appraisal | The Mixed Methods Appraisal Tool (MMAT). | Global unweighted score based on critical appraisal to grade studies - referenced to Hawker et al. Articles with seven or more of the nine criteria were defined as high-quality studies. 4-6 medium quality. Risk of Bias. | None reported | Individual appraisal: Johanna Briggs Institute Qualitative Assessment and Review Instrument (JBI-QARI). Cross-study comparative appraisal: GRADE-CERQual. | Risk of bias: MMAT Version 2018. Risk of bias in the overall body of evidence: qualitatively evaluated via discussion. | The Joanna Briggs Institute critical appraisal checklists. |
| Population | Patients and carers | Unspecified | Cancer patients | Older persons > 65 years of age | 18 years or older with any stage of cancer | Older persons 60 years or older |
| Inclusion criteria | Studies of all patients/clients/users/ and their carers (male and female) of health and social care services of any age that included patient or carer reported experience after the introduction of the Health and Social Care Act. Studies that focused on integrated care (defined as changes in integration/coordination of service delivery in order to improve patient outcomes and experience) involving health, social and/or third sector organisatins within UK. Any study design, English language, empirical studies published in peer reviewd journals. | Empirical quantitative or qualitative research investigating the role of hospitals in the care of chronically ill patients. | English or Italien; Abstract available online for review; Abstracts describing cancer patients (in any step across the care pathway including screening, diagnosis, active treatment, follow-up, palliative care, post-treatment survivorship) and integrated care (or related concepts), and patient experience (or related concepts). | Studies using qualitative methods, a semi-structured or open-ended questioning approach; exploring older persons’ self-reported experiences with relevance to the research topic; experiences of persons aged 65 or over adapting to life at home after hospital discharge. Original research, including peer-reviewed articles and doctoral theses, were included. | Peer-reviewed randomized controlled studies, non-randomized controlled studies, quantitative descriptive studies, or qualitative interviews or focus groups; Inclusion of patients aged 18 years or older diagnosed with any type or stage of cancer; Inclusion of patients with one or more chronic comorbid condition, of any kind; Inclusion of patient perceptions, experiences, or needs related to care coordination between PCPs and oncologists; Ability to extract results | Published peerreviewed studies and grey literature reporting on the views of older adults aged ≥60 years (male or female) who had received integrated or similarly coordinated care of any definition in any type of healthcare setting. |
| Exclusion criteria | Integrated care which focused on acute care episodes only, e.g. emergency care models or surgical pathways. Experience consisted of satisfaction score only. Studies which did not adress the quality issues specific to this review with sufficient depth. Therefore, were unable to add value to findings. | Articles unrelated to hospitals, theoretical and conceptual analysis, abstracts of meetings, review articles, editorials, letters. Studies set in community or hospice settings, psychiatric care, or children's care. Stduies investigating or describing individual hospital programs without accentuating the "integration" factor. | No focus on research questions | Not reported | Study design criteria not met, did not include care strategies of PCP/oncologist, Not peer reviewed, not focused on cancer patients, patients < 18 years old, no inclusion of patient perceptions of care coordination. | None reported. Flowchart full text screening: Not about older people; Participant voice not represented; Not relevant to integrated care; Full text unavailable online. |
| Initial number of studies | 23731 | 11220 | 1760 | 645 | 153 | 336 |
| Included number of studies | 16 | 32 | 30 | 13 | 22 | 30 |
| List included studies by author and publication year | Boudioni 2015, Darwin 2017, Deslandes 2015, Fairbrother 2013, Firn 2018, Gowing 2015, Hamilton 2016, Hu 2014, Mastellos 2014, Moule 2014, Payne 2017, Primeau 2017, Randall 2014, Thomson 2018, Wye 2014, Young 2019. | Abad-Corpa 2013, Akosah 2002, Atienza 2004, Baldwin 2014, Blue 2001, Brand 2004, de la Porte 2004, Chiu 2001, Cline 1998, Coleman 2004, Coleman 2006, Cowie 2009, Dossa 2012, Farrero 2001, Grunfeld 1999, Hanumanthu 1997, Harrison 2002, Ireson 2009, Jeansawang 2012, Ledwidge 2005, Linden 2014, Luttik 2014, Moalosi 2003, Naithani 2006, Naylor 2004, Rauh 1999, Ricauda 2008, Sadatsafavi 2013, Shi 2015, Vliet Vlietland 1997, Williams 2003, Williams 2010. | August 1995, Birchall 2002, Boman 1999, Campbell 2010, Carroll 2010, Chen 2010, Drury 2010, Ell 2006, Fillion 2009, Fillion 2012, Fiscella 2009, Gilbert 2011, Goodwin 2003, Gosh 2001, Guadagnolo 2011, Komatsu 2011, Korber 2011, Lee 2011, Lilliehorn 2010, Lundstrøm 2011, Nielsen 2003, O'Brien 2010, Pieters 2011, Rodriguez 2011, Sahay 2000, Swanson 2010, Wagner 2010, Walker 2003, Walsh 2010, Wolfe 1993. | Andreasen 2015, Bagge 2014, Dilworth 2012, Dossa 2012, Jones 2012, Karlsson 2016, Knight 2011, McKeown 2007, Neitherman 2015, Perry 2011, Reay 2015, Rydeman 2008, Slatyer 2013. | Arora 2011, Aubin 2012, Boekhout 2015, Brennan 2016, Cheung 2009, Durcinoska 2017, Duthie 2017, Fenlon 2013, Geller 2014, Goebel 2016, Grunfeld 2011, Haggstrom 2009, Hudson 2012, Krishnasamy 2011, Mao 2009, Palmer 2015, Parry 2011, Rutledge 2017, Wagner 2010, Wallner 2017, Walsh 2011, Wieldraaijer 2018. | Algilani 2017, Baillie 2014, Bayliss 2008, Berendsen 2016, Blom 2016, Burridge 2016, Cheng 2017, Cook 2017, Cowie 2009, Derksen 2012, Ebrahimi 2017, Freeman 2010, Hepworth 2013, Jackson 2012, Jeon 2010, Johnston 2009, Jubelt 2014, Liss 2011, National Voices 2012, National Voices 2013, Osborn 2014, Rimmer 2015, Roland 2012, Ryan 2013, Sada 2011, Sharma 2014, Stevens 2014, Toscan 2012, Vat 2015, Wodskou 2014. |
| Qualitative themes | Seven themes: (I) Coordinated care within teams; (II) Coordinated care across team; (III) Coordinated across team and community services; (IV) Continuous familiarity over time; (V) Continuous proactive and responsive action between visits; (VI) Patient centered care; (VII) Shared responsibility. | Six themes: (I) Longitudinal continuity; (II) Relational continuity; (III) Flexible continuity; (IV) Team and cross-boundary continuity; (V) Information; (VI) Trust. | Aspects of integrated care: (I) Individual care provider, team care providers, mixed-approach; (II) Continuity of care; (III) Empowerment; (IV) Coordination; (V) Information; (VI) Shared decision making; (VII) Accessibility of services; (VIII) Needs support. | Three themes: (I) Unsafe transition (Information, medication, involvement, errors, communication between providers); (II) New situation at home (Dependent on help from others, independence, care according to needs); (III) Paternalistic model (Personnel distant/stressed, Being seen or heard, involvement). | Four themes: (I) Communication; (II) Defining provider care roles and expectations; (III) Patients' need for easy access to care information; (IV) Individualized patient care. | Five themes: (I) Access and availability; (II) Involvement, initiative and follow-up; (III) Communication and information; (IV) Referral and care transitions; (V) Coordination and cooperation. |

| Title | Survivors’ preferences for the organization and delivery of supportive care after treatment: An integrative review | Measuring Patients’ Experience of Rehabilitation Services Across the Care Continuum. Part II: Key Dimensions | The challenges of uncertainty and interprofessional collaboration in palliative care for non-cancer patients in the community: a systematic review of views from patients, carers and health-care professionals. | The experience of discharge for patients with an acquired brain injury from the inpatient to the community setting: A qualitative review | Patients' perspectives on the medical primary-secondary care interface: systematic review and the synthesis of qualitative research | An integrative model of patient-centeredness - a systematic review and concept analysis |
| --- | --- | --- | --- | --- | --- | --- |
| Author(s) | Samantha J. Mayo, Rand Ajaj, Amanda Drury | Josephine McMurray, Heather McNeil, Claire Lafortune, Samantha Black, Jeanette Prorok, Paul Stolee | Ai Oishi, Fliss EM Murtagh | Loretta Piccenna, Natasha A. Lannin, Russell Gruen, Loyal Pattuwage, Peter Bragge | Rod Sampson, Jamie Cooper, Rosaline Barbour, Rob Polson, Philip Wilson | Isabelle Scholl, Jördis M Zill, Martin Härter, Jörg Dirmaier |
| Year of publication | 2021 | 2016 | 2014 | 2016 | 2015 | 2014 |
| Countries of origin | Australia (7), Belgium (1), Canada (11), China (2), Denmark (1), Ireland (1), Japan (1), Korea (1), Netherlands (9), Norway (1), Spain (1), United Kingdom (5), USA (28) | Australia (3), Canada (1), England (2), France (2), Germany (2), Ireland (1), Italy (2), Korea (1), Netherlands (3), Norway (2), Scotland (2), Spain (3), Sweden (2), Switzerland (1), USA (6) | Australia, New Zealand (2), Sweden, United Kingdom (25), USA | Australia (5), Brazil, Canada, United Kingdom, USA | Australia, England (10), Israel, Netherlands (2), New Zealand, Scotland (2), Sweden. 2 studies included Italy, Netherlands, Poland, Spain and Sweden. | Asian countries (12), Australia and New Zealand (17), Canada (27), Germany (27), Netherlands and Belgium (21), Other European Countries (13), Scandinavian Countries (13), South Africa (2), UK and Ireland (62), USA (223) |
| Setting | Post-treatment supportive care service programs for cancer. | Rehabilitative care system | Palliative care by PCPs | Inpatient to community setting | Primary-secondary care interface | None specified |
| Aim/purpose | To map the literature regarding survivors’ preferences for the organization and delivery of supportive care after treatment to inform healthcare professionals of these preferences, identify evidence gaps, and suggest areas of future research. | To identify major themes related to the measurement of patient experience of care across the rehabilitative care system. | Identifies, critically appraises and synthesizes the existing evidence on views on the provision of palliative care for non-cancer patients by PCPs and reveals any gaps in the evidence. | To provide a synthesis of the perspectives of people with ABI, representatives, caregivers, families or relatives on the transition from hospital to home. The study objectives were to: (1) conduct a systematic qualitative literature search post-dating that of the previous published review (i.e. from 2007 onwards); (2) identify key themes which may inform further research efforts; and (3) evaluate key themes from identified studies in the context of those previously elucidated. | To identify what patients perceive as important markers of care quality at the primary-secondary care interface. | To identify and analyze the different dimensions of patient-centeredness described in the literature. To propose an integrative model of patient-centeredness. |
| Study design | Integrative review | Systematic review | Systematic review | Qualitative review | Systematic review | Systematic review |
| Theoretical framework and/or additional key features | Deductive content analysis. The coding structure was guided by thematic categories of organization of survivorship services identified in an environmental scan by Howell. | Thematic analysis with multiple levels of coding using a constant comparative method. | The Centre for Reviews and Dissemination’s guidance for undertaking reviews in health care. PRISMA. Supplemented by guidance on narrative synthesis. | Not reported | A meta ethnographic approach described by Noblit and Hare. Enhancing transparency in Reporting the synthesis of qualitative research (ENTREQ). PROSPERO. | Conventional content analysis. |
| Search databases | PUBMED, CINAHL, PsycINFO | MEDLINE (PubMed), CINAHL (EBSCO), PsycINFO (APA PsycNET). Journals: Healthcare Policy, WHO Bulletin, Health Affairs, International Journal for Quality in Health Care. | MEDLINE, Embase, CINAHL, PsycINFO, Applied Social Science Abstract and the Cochrane library, Cited refrence serach in SCOPUS. Journal: Palliative Medicine. Grey: CareSearch, Open Grey | MEDLINE (R) In Process & Other Non-Indexed Citations and MEDLINE(R) (OvidSP), all EBM (Cochrane Database of Systematic Reviews, ACP Journal Club, Database of Abstracts of Reviews of Effects, Cochrane Methodology Register, Health Technology Assessment, NHS economic evaluation database and the Cochrane Central Register of Controlled Trials), CINAHL, PsycINFO, and Web of Science. Google Scholar. | EMBASE (OVID), MEDLINE (OVID MEDLINE), CINAHL Plus with Full text (EBSCO host), PsycINFO, Psychology and Behavioural Sciences Collection, Health Business Elite, Biomedica Reference Collection: Comprehensive Library, Information Science & Technology Abstracts, eBook Collection (EBSCO host), Web of Science Core Collection: Citation Indexes and Social Sciences Citation Index, and grey literature sources: Open SIGLE, Health Management Information Consortium, National Technical Information Service and PsycEXTRA. | MEDLINE, EMBASE, PsycInfo, Cochrane Library, Psyndex. |
| Key search terms | ‘Cancer Survivor’, ‘Patient Preferences’, ‘Service Delivery’ and ‘Supportive Care’ | Rehabilitation, physical therapy, or occupational therapy AND Patient experience, patient centered care, or patient satisfaction. | Group 1: Disease diagnoses; Group 2: ‘Palliative care’; Group 3: ‘Primary care’; Group 4: ‘Attitude’ | ‘Brain injury’ combined with ‘patient perception of transition of care from hospital to home’ | Variations of (Family practice OR general practice OR primary care) AND (Secondary care OR hospitals OR consultants) AND (Qualitative research OR Patient satisfaction OR patient experience) | "Patient-centered" or "Patient-centeredness". Did not include synonyms eg. "patient-focused" or "person-centered". |
| Search dates/years | Past 10 years. Search conducted May 2019, updated September 2019 | 2004 - 2014 | Inception to September 2012 | January 2007 - May 2015 | Inception to 30th July 2014 (Grey 7th August 2014) | Inception to January 2012 |
| Languages | English | English | English | Not reported | None applied | Not reported |
| Quality appraisal | None reported | None reported | Amended Hawker’s criteria. | The Critical Appraisal Skills Programme (CASP) Qualitative Research Checklist. | The CASP tool was applied post-inclusion. | No |
| Population | Adult survivors of cancer excluding adult survivors of pediatric cancer. | Patients recieving rehabilitative care | Patients, carer and health care professionals in primary palliative care. | Patients with acquired brain injury (ABI), carers and HCPs. | Patients | Not reported |
| Inclusion criteria | PEOS; Participant: Cancer survivors with a diagnosis of any malignancy, aged 18 years or older who have completed primary treatment; Exposure: Cancer survivors who have completed active cancer treatment and are living with or after cancer. Outcomes: We considered survivors’ preferences for the organization  and structure of supportive care delivery in the post-treatment  period. Study: Primary non-experimental research studies, inclusive of quantitative, qualitative, and mixed methods study designs. | Articles must have been published in a peer-reviewed journal in the English language since 2004. A direct and obvious relevance to rehabilitative care, were patient experience focused, and discussed a specific measurement tool that had been empirically tested. Only those articles where the questions were appended to the article, or the questionnaire was publicly available to review, were included. | SPIDER. Sample: Patients, carers or professionals. Studies with patients or carers as participants  are considered when 50% or more of the participants or the  ones they cared for had non-cancer diagnoses; Phenomenon  of interest: Primary palliative care provision to non cancer patients at home; Design: Any study designs, both quantitative  and qualitative; Evaluation: Participants’ views on this phenomenon. | Study type: Qualitative study defined as ‘developing explanations of social phenomena’ that aim to seek answers to the ‘why, what and how’ of the phenomena. Mixed methods studies were included if they had a primarily qualitative focus; Study participants: Patients with an acquired brain injury (ABI) and/or patient representatives, families and caregivers (formal or informal/unpaid) of those affected by an ABI. | Primary studies; Employed qualitative methodology; Explored patients' perspectives; Targeted the medical primary-secondary care interface; Full research papers. | Publications, which had a conceptual definition of patient-centered care were eligible for inclusion. A definition was conceptual if it specified "what needs to be assessed in empirical evidence". |
| Exclusion criteria | Studies focusing on adult survivors of paediatric cancer or caregivers of adult survivors; Outcomes that focused directly on survivors’ needs or others, if not directly related to preferences for care delivery; Conference proceedings, case reports, pilot or feasibility studies and intervention studies; Studies published prior to 2009 or in a language other than English were excluded. | Articles addressing mental health, palliative care, pediatrics, dental, or veterinary rehabilitation. Qualitative study designs were excluded from the review but were maintained to inform the discussion section of the article. | None reported | Studies were excluded if patients had conditions other than ABI; if there was no direct engagement with patients and/or caregivers and/or if they examined the transition between inpatient care settings. | Non-qualitative methodology; Did not explore patients' perspectives; Did not focus on the medical primary-secondary care interface; Not full research papers | Records that were short commentaries, conference abstracts, book reviews, letters to editors etc. |
| Initial number of studies | 744 | 2442 | 3986 | 4067 | 654 | 2660 |
| Included number of studies | 69 | 33 | 31 | 10 | 20 | 417 |
| List included studies by author and publication year | Absolom 2009, Andersen 2009, Arch 2018, Baena-Cañada 2013, Beehler 2017, Bender 2012, Bender 2016, Bessen 2014, Boyajian 2014, Brennan 2011, Burke 2016, Cheung 2009, Crouch 2017, de Rooij 2018, den Bakker 2018, Denzen 2019, Dyer 2016, Garland 2018, Gjerset 2011, Hudson 2012, Hugh-Yeun 2017, Huibertse 2017, Jansen 2015, Karvinen 2011, Khan 2011, Kwan 2019, Lawn 2017, Lee 2016, Li 2018, Llewllyn 2019, Lobb 2009, Lubberding 2015, Martinez Tyson 2017, McCallum 2014, Meade 2017, Monterosso 2017, Monterosso 2018, Nicolaije 2013, Oskam 2013, Park 2018, Parry 2011, Pauwels 2013, Philip 2016, Phillips 2017, Piil 2018, Psihogios 2019, Pugh 2018, Rabin 2013, Ramsay 2018, Sanders 2010, Saroa 2018, Sclumbrecht 2018, Shea-Budgell 2014, Singh-Carlson 2013, Smith 2011, Smith 2019, So 2019, Sporn 2015, Tarver 2019, Tompkins 2016, Tucholka 2018, Umezawa 2015, Urquhart 2012, van de Poll-Franse 2008, Wallner 2017, Wen 2014, Wieldraaijer 2018, Wilkins 2012, Zhang 2017. | Almborg 2009, Baumann 2011, Beattie 2002, Beattie 2005a, Beattie 2005b, Cott 2006, Cramm 2012, Curry 2012, Delanian 2011, Dibbelt 2009, Duijin 2008, Elwood 2010, fernandez 2007, French 2010, Grosset 2005, Grotle 2009, Hills 2007, Hush 2013, Kneebone 2012, Knight 2010, Medina-Mirapeix 2012, Medina-Mirapeix 2013a, Medina-Mirapeix 2013b, Monnin 2001, Normann 2012, Ottonello 2012, Quaschning 2013, Roush 2007, Slade 2010, Soderback 2008, Stubbe 2007, Van der Linde 2007, Vanti 2013 | Skilbeck 1998, Oliver 2001, Jones 2004, Gysels 2008, Shipman 2009, Brumley 2007, Seamark 2004, Edmonds 2007, Whitehead 2011, Pinnock 2011, Murray 2002, Boyd 2004, Boyd 2009, Waterworth 2011, Hughes 2005, Exley 2005, Fitzsimons 2007, Elkington 2004, Herz 2006, Addington-Hall 1995, Young 2008, Hasson 2010, McLaughlin 2011, Disler 2010, Hanratty 2002, Hanratty 2006, Waterworth 2010, Brännström 2011, Grisaffi 2010, Field 1998 | Almborg 2009, Bauer 2011, Fitzgerald 2011, Gustafsson 2013, Keightley 2011, Mackenzie 2007, Rodrigues 2013, Rotondi 2007, Turner 2007, Turner 2011. | Admi 2013, Bain 2000, Bain 2002, Beech 2013, Berendsen 2009, Burkey 1997, Davies 2006, Flink 2013, Göbel 2012, Hesselink 2013, Hesselink 2012, Kemp 2013, McHugh 2007, Pascoe 2013, Pollard 2011, Preston 1999, Somerset 1999, Walton 2013, Wilkes 2009, Wright 2006 | All 417 studies not listed |
| Qualitative themes | Five themes: (I) Accessibility, convenience and flexibility of care; (II) Care appropriately balanced by the level of intensity required; (III) Support from professionals with specialized expertise in the area of need was often preferred; (IV) Information that supports self-management; (V) Being able to access credible sources of information. | Five themes: (I) Rehabilitative care ecosystem; (II) Client and informal caregiver engagement; (III) Patient and health care provider relation; (IV) Pain and functional status; (V) Group and individual identity. | Ten themes: (I) GPs as partners; (II) Quick responses to urgent needs; (III) Continuity of care and regular monitoring; (IV) Communication between care providers; (V) Unclear boundaries of the roles of professionals; (VI) Access; (VII) Information; (VIII) Coordination of care; (IX) Fear of bothering HCPs inappropriately; (X) Expertise. | Two main themes: (I) Engagement; (II) Support. Three sub-themes: (I) Poor communication; (II) Limited participation; (III) Disorganized arrangements for support services. | Four themes: (I) Barriers to care; (II) Communication; (III) Coordination; (IV) Relathionships and personal values. | Three themes: (I) Principles: Essential characteristics of the clinician, clinician-patient relationship, patient as a unique person, biopsychosocial perspective; (II) Enablers: Clinician-patient communication, integration of medical and non-medical care, teamwork and teambuilding, access to care, coordination and continuity of care; (III) Activities: Patient information, patient involvement in care, involvement of family and friends, patient empowerment, physical support, emotional support. |

| Title | Patient-perceived health service needs in inflammatory arthritis: A systematic scoping review | Patients' and healthcare providers’ perspectives on diabetes management: A systematic review of qualitative studies | The Warwick Patient Experiences Framework: patient-based evidence in clinical guidelines | Continuity of care and quality care outcomes for people experiencing chronic conditions: A literature review | What do we know about patients’ perceptions of continuity of care? A meta-synthesis of qualitative studies | Mapping Evidence of Patients’ Experiences in Integrated Care: A Scoping Review |
| --- | --- | --- | --- | --- | --- | --- |
| Author(s) | Julian D. Segan, Andrew M. Briggs, Louisa Chou, Kathryn L. Connelly, Maheeka Seneviwickrama, Kaye Sullivan, Flavia M. Cicuttini, Anita E. Wluka | Phoutsathaphone Sibounheuang, Phayom Sookaneknun Olson, Pattarin Kittiboonyakun | Sophie Staniszewska, Felicity Boardman, Lee Gunn, Julie Roberts, Diane Clay, Kate Seers, Jo Brett, Liz Aviatl, Ian Bullock, Norma O’ Flynn | Gwen van Servellen, Marie Fongwa, Ellen Mockus D’Errico | Sina Waibel, Diana Henao, Marta-Beatriz Aller, Ingrid Vargas, María-Luisa Vázquez | Alaa Youssef, Zarah K. Chaudhary, David Wiljer, Maria Mylopoulos, Sanjeev Sockalingam |
| Year of publication | 2018 | 2020 | 2014 | 2006 | 2012 | 2019 |
| Countries of origin | Australia, Canada, Estonia, Japan, Netherlands (3), Sweden, Switzerland, United Kingdom (13), USA (5) | American Samoa, Australia, Bangladesh, Canada (3), Finland, Germany, Iran, Japan, Malaysia, Netherlands (2), Oman (2), Taiwan, United Kingdom, USA (6) | Not reported | Australia (3), Canada (3), Denmark, Finland, Netherlands (4), Norway, Spain, Sweden, United Kingdom (5), USA (12) | Australia (2), Belgium, Canada (4), Sweden, United Kingdom (15), USA (2) | Australia, Canada,India, Netherlands, New Zealand, United Kingdom (5), USA (11) |
| Setting | Inflammatory arthritis care | Diabetes management | Not reported | Not reported | Not reported | Integrated care settings |
| Aim/purpose | To identify and synthesise the existing literature regarding patient-perceived health service needs relating to inflammatory arthritis. To identify evidence where patients’ perspectives, captured either quantitatively or qualitatively, were reported, so as to provide a reliable summary of patient self-report data. | This study aimed to explore the perspectives of both health care providers and diabetic patients utilizing the CCM as an analytical framework in order to gain more in-depth information regarding their contexts as well as to identify factors that can help to improve the provision of diabetes care services. | Sampled a range of patient experiences studies, with the intention of reaching a level of data saturation, in terms of the generic themes being identified for each group. The intention was to use this concept of data saturation to identify all relevant generic themes from the evidence reviewed. | (i) What is the frequency of studies examining the relationship of continuity of care and quality of care in the clinical trial literature and in nursing journals in particular?; (ii) Which types and combinations of continuity of care strategies are addressed in this literature?; (iii) Which quality of care indicators are associated with continuity of care-enhancing strategies or programs?; (iv) Which recommendations for future research emanate from this literature review that addresses the relationships between continuity of patient care and quality care outcomes? | What are patient’s views on RC, IC and MC across care levels? What is their attributed relevance? What are the causes and consequences of perceived discontinuity? | The primary research question aimed to examine the breadth of existing evidence on patients' experience with chronic illness receiving care in IC settings; the secondary research question focused on identifying the dimensions of patient experience that were affected by IC setting structural components and care context. |
| Study design | Scoping review | Systematic review | Scoping review | Systematic review | Meta synthesis | Scoping review |
| Theoretical framework and/or additinal key features | A scoping review approach as described by Arksey and O’Malley, underpinned by systematic review principles for evidence identification and analysis. The principles of meta-ethnography were used to synthesise the data. | The Chronic Care Model (CCM) was used as analytical framework. Thematic analysis. | Inductive analysis until data saturation. Comparison to IOM framework. | Systematic review based upon the guidelines adopted by Sparbel and Anderson. Narrative analysis. | Findings were separated by type and dimension of continuity, according to the theoretical framework by Reid et al. The analytical process largely followed the classic method of Noblit and Hare. | The Arksey and O'Malley scoping review framework, enhanced by Levac et al. The Joanna Briggs Institute (JBI) scoping review guidelines. PRISMA prior to the development PRISMA-ScR. Thematic analysis. |
| Search databases | CINAHL, EMBASE, MEDLINE, PsycINFO | PubMed (Medline), Science Direct, Web of Science, CINAHL | Medline, Cinahl, Assia, Embase, Psychinfo. Additional papers were identified from reference lists and specialist journals. Additional searches were carried out on PubMed and UK PubMed Central. | Medline | Medline, Social Sciences Citation Index and Science Citation Index Expanded | MEDLINE, EMBASE, Psyc INFO, CINAHL, AMED and the Cochrane Library. Grey literature from Google Scholar, Web of Science and key research journals. |
| Key search terms | Combining both MeSH terms and text words to capture evidence regarding patients’ perceived health service needs (defined as self-reported expectations, desires or requirements) relating to inflammatory arthritis. | Keywords and strategies were ‘Type 2 Diabetes Management’ used with a Boolean ‘AND’ to conjugate with the following words ‘Patient needs,’ ‘Patient perceptions,’ ‘Patient opinions,’ ‘Patient perspectives,’ ‘Provider needs,’ ‘Provider perceptions,’ ‘Provider opinions,’ and ‘Provider perspectives’ |  | The general subject heading was “continuity of patient care.” An additional subject heading guided the and included “continuity of patient care and quality of care.” | The search strategy included the combination of descriptors and keywords relating to the research area (‘continuity of care’ or linked key terms that were similar in meaning), qualitative characteristics and the patient’s perspective, utilizing the Boolean operator ‘AND’. | Collaboration, coordination, integration, or chronic care model AND patient experience, centered, satisfaction, perspective, perception, participation, or engagement. OR empathy, compassion, respect, responsiveness, kindness, or dignity. |
| Search dates/years | January 1990 - July 2016 | January 2001 - September 2017 | 1995 - 2011 | 1 January 1996–1 June 2005 | Until 2009 | 1988 - August 2018 |
| Languages | English | English | English | English | English, German or Spanish, |  |
| Quality appraisal | Qualitative studies were assessed using the CASP tool. Quantitative studies were assessed using a modified incidence/prevalence study tool designed to identify bias created by Hoy et al. | The CASP for qualitative research was used and assigned a value point. Articles included for the study were required to have at least five points. | Not reported | Not reported | Mays and Pope’s criteria of validity | Not reported |
| Population | Patients with inflammatory arthritis | Patients with T2DM and health care providers | Adult patients' perspectives in cardiovascular disease, diabetes and cancer | Not stated | Patient perspectives | Patients perspectives |
| Inclusion criteria | (1) Studies had to concern patients older than 18 with rheumatoid arthritis or other inflammatory arthritis; (2) Studies had to report on patients’ perspective (3) studies had to relate to patients’ perceived needs, encompassing expectations, desires and requirements related to non-pharmacologic health services, including conventional medicine, allied health and complementary and alternative medicines and therapies (CAM); (4) Studies had to concern inflammatory arthritis, predominantly. | The authors began by examining studies that were (1) qualitative studies involving T2DM patients and, (2) Studies, which aimed to evaluate patients' and providers’ perspectives on diabetes management. All studies were available in full-text format with the quotations shown in the result part of included articles. CASP minimum 5 points. | Research papers that focus on exploring or identifying patient experiences in adult services in three clinical areas: cardiovascular disease, diabetes and cancer. English language papers. ‘Search dates’: 1995–2011. | Clinical trials; Continuity of care | (i) Relevance to the research topic (explicitly or implicitly analysing RC, IC or MC), (ii) original studies that adopted a qualitative design and (iii) investigating the patient’s perspective. T | Peer-reviewed journal papers eligible for inclusion met the following criteria: published between the period of 1988-Aug 2018, written in English language, examined patient experience in IC settings. In this review, we conceptualized integrated care as a continuum of care that comprise a range of care models based on differences in clinical setting structure and care team-coordination level. Therefore, IC studies eligible for inclusion in this scoping review had to meet the following inclusion criteria: 1) meet SAMHSA (CIHS) integration framework description in regard to IC clinical setting structure/level of coordination (Appendix 2) align explicitly with the chronic care model and collaborative care core principles defined in the APA-APM Dissemination of Integrated Care 2016 Report. |
| Exclusion criteria | None reported | (1) trialed an intervention in the study (e.g. technology, program, training, education), (2) studied special groups of patients such as immigrants or disabled patients, or examined special events such as Ramadan or travels, or (3) described other perspectives such as that of family members, or people who were only at risk for T2DM | Papers that primarily focus on interventions to enhance patient experiences. Papers that report development, testing or application of patient-reported outcome measures. Opinion articles or editorials about patient experience. NonEnglish language papers. Children’s experiences. Carer’s experiences. Grey literature | The remaining eight were dropped for the following reasons: continuity of care was treated as the only dependent variable in two articles, only the design and intervention without the results were offered in three articles, the target population was either the family or professional caregiver and the effects on the patient were not reported in two papers, and the intervention focused only upon the recruitment of patients to the study in one paper. | Not reported | We excluded studies examining patient experience using single selfreported measures (i.e. PACIC) due to inconclusive evidence and nonstandardized application to evaluate patient experience in IC. |
| Initial number of studies | 1218 | 1981 | ? | ? | 601 | 5250 |
| Included number of studies | 27 | 23 | ? | 32 | 25 | 21 |
| List included studies by author and publication year | Ahlmen 2005, Arthur 2004, Berkanovic 1995, Bhutani 2013, Blake 2013, Buchbinder 2002, Douglas 2005, Dures 2016, Feldman 2010, Flurey 2013, Fraenkel 2014, Hughes 2009, Ikuyama 2009, Jong 2012, Kumar 2010, Lempp 2006, Niedermann 2010, Nota 2016, Oliver 2008, Pollard 2011, Polluste 2012, Radford 2008, Rao 1998, Rao 1999, Rose 2006, Van der Haart 2014, Ward 2007. | Abdulhadi 2012, Alazri 2010, Al-Azri 2011, Al-Quazaz 2011, Beverly 2011, Beverly 2012, Brez 2009, Brown 2002, Carbone 2006, Dhippayom 2015, Dutton 2014, Gerda Längst 2015, Goetz 2012, Halkoaho 2013, Held 2010, Kato 2016, Kern 2001, Lai 2006, Lewis 2014, Matthews 2008, Moonaghi 2014, Raaijmakers 2013, Wen An Huang 2005. | Not reported | Atienza 2004, Byng 2004, Cowan 2004, Crotty 2004, Druss 2001, Evans 2997, Fagerberg 2000, Fjaertoft 2004, Grunfeld 1999, Harrison 2002, Keitz 2001, Moher 2001, Naylor 2004, Neilsen 2003, Preen 2005, Reynolds 2004, Roy-Byrne 2001, Samet 2003, Simon 2002, Smeenk 1998, van Eaton 2005, van der Weide 1999, Williams 2001 - not all are listed. | Alazri 2006, Boulton 2006, Campbell 2007, Cowie 2009, Green 2008, Guthrie 2008, Harrison 2002, Infante 2004, Jones 2009, Lester 2005, McCormack 2008, Mercer 2007, Michiels 2007, Nair 2005, Naithani 2006, Nazareth 2008, O'Cathain 2008, Pandhi 2007, Pooley 2001, Preston 1999, Von Bültzinglöwen 2006, Williams 2004, Woodward 2004, Wong 2008, Wong 2009. | Balasubramanian 2017, Batka 2016, Bauer 2018, Brener 2013, Davis 2018, Knowles 2015, Mead 2014, Mukherjee 2006, Nadeau 2017, Noël 2004, Ohl 2013, Rao 2016, Saini 2016, Sheff 2017, Simpson 2008, Small 2017, Spoorenberg 2015, Stanhope 2014, Treloar 2014, Webster 2016, Wrenn 2017. |
| Qualitative themes | Two main themes: (I) Communication: Empathetic and positive attitude, understanding, clear communication, being involved in decision-making; (II) Aspects of care: Length of consultation, timely care, accessible review, specialist referral, and in times of need. | Eight themes: (I) Community linkage; (II) Healthservice systems; (III) Continuity of care; (IV) Self-management; (V) Effective health care providers; (VI) Referral system; (VII) Patient-provider interaction; (VIII) Family involvement. | Thirteen themes: (I) Compassion, empathy and responsiveness; (II) Co-ordination and integration; (III) Information, communication and education; (IV) Physical comfort; (V) Emotional support, relieving fear and anxiety; (VI) Involvement of family and friends; (VII) Patient as active participant; (VIII) Responsiveness of services - an individualized approach; (IX) Lived experience; (X) Continuity of care and relationships; (XI) Communication; (XII) Information; (XIII) Support | Seven themes: (I) Provider influence; (II) Patient satisfaction, well-being, and unmet needs; (III) Health status, symptom severity, symptom relapse, and worsening condition or mobility; (IV) Adverse events; (V) Informational continuity; (VI) Relational continuity; (VII) Management continuity. | Main theme: Patient involvement or participation, including personal self-responsibility, are critical to continuity. Management Continuity: (I) Accessibility to different levels of care; (II) Smooth discharge process; (III) Individualized care. | Cross-cutting theme: the way that therapeutic spaces are designed can influence the way that patients experience their care: (I) Personalizing care to address individual needs; (II) Developing collaborative care relationships; (III) Facilitating access to care. |

| Title | The meaning of continuity of care from the perspective of older people with complex care needs - A scoping review | The Experience of Chronic Illness Transitional Care: A Qualitative Systematic Review | Indigenous Australians’ Experiences of Cancer Care: A Narrative Literature Review |  |  |  |
| --- | --- | --- | --- | --- | --- | --- |
| Author(s) | Ingrid Djukanovic, Amanda Hellström, Anna Wolke, Kristina Schildmeijer | Jee Young Joo, Megan F. Liu | Saira Sanjida, Gail Garvey, James Ward, Roxanne Bainbridge, Anthony Shakeshaft, Stephanie Hadikusumo, Carmel Nelson, Prabasha Thilakaratne and Xiang_Yu Hou |  |  |  |
| Year of publication | 2024 | 2022 | 2022 |  |  |  |
| Countries of origin | USA (3), Canada (3), Sweden (4), Norway (2), the Netherlands, Australia, the UK (2), Germany and Denmark. | Australia (2), Canada, Netherlands, Norway, Turkey, USA | Australia |  |  |  |
| Setting | Care continuum | Hospital-to-home transitions | Cancer care continuum |  |  |  |
| Aim/purpose | How is continuity of care framed in the context of older people with complex care needs? In what way is continuity of care significant for older people with complex care needs? In what care situations is continuity of care emphasised by older people with complex care needs? What factors affect the continuity of care for older people with complex care needs? | What experiences and perceptions with transitions from healthcare facilities to other settings did individual(s) with chronic condition(s) and their caregivers have? | What are the experiences among Indigenous people with cancer when they go through different sectors of the healthcare system in Australia? |  |  |  |
| Study design | Scoping review | Qualitative systematic review | Narrative review |  |  |  |
| Theoretical framework and/or additinal key features | A scoping review, based on the framework from Arksey and O’Malley as refined by Levac et al. was chosen as the study design | The qualitative systematic review methodology with thematic synthesis was applied (Thomas & Harden, 2008). The PRISMA Statement and PICO framework were used | An inductive approach was used to qualitatively evaluate individual studies’ themes and quotations |  |  |  |
| Search databases | PubMed, Cinahl, PsycInfo, ASSIA, Web of Science, Google Scholar, Scopus, and DOAJ. For grey literature, the database BASE was used, and a hand-search of two key journals was performed to broaden the search and to not miss literature that is not indexed in traditional databases. The International Journal of Integrated Care and Journal of Comorbidity were searched via their tables of content, and texts published in 20142020 and written in English were included | MEDLINE, CINAHL, PsycINFO, and Web of Science | PubMed, CINAHL, and Scopus. Hand search of included papers’ reference lists |  |  |  |
| Key search terms | Combining both MeSH terms and text words to capture evidence regarding patients’ perceived health service needs (defined as self-reported expectations, desires or requirements) relating to inflammatory arthritis. | Key terms and MeSH were combined with operators: “transition” or “transitional care” or “transitional care management” or “transitional care service” or “transitional care model”) and (“qualitative study” or “qualitative research” and (“chronic illness” or “chronic condition” or “chronic disease”). Additional keyword searches: experience or perception; discharge, or discharge planning; carer or caregiver or patient; challenge, or barrier; grounded theory, or ethnography, or phenomenology; or nurse, or healthcare provider, or healthcare practitioner | Main keywords and Medical Subject Headings (MeSH) search terms were ‘Indigenous people’ AND ‘cancer’ AND ‘healthcare service’ AND ‘Australia’ AND ‘community’ AND ‘health service’ |  |  |  |
| Search dates/years | January 2014 – April 2023 | January 1, 2012 - June 30, 2021 | Inception to June 2022 |  |  |  |
| Languages | English | English | English |  |  |  |
| Quality appraisal | No quality assessment | The Critical Appraisal Skills Programme (2013) Qualitative Research Checklist was used to check the qualitative studies’ methodological quality. | Not reported |  |  |  |
| Population | Older people with complex care needs | Adult patients with at least one chronic illness | Indigenous people of Australia living with cancer and carers |  |  |  |
| Inclusion criteria | Inclusion criteria for the review were as follows: (1) Age 65 with at least one chronic condition, (2) the patient’s experience of continuity of care, and (3) any type of primary studies and documents (quantitative, qualitative, or mixed methods, grey literature) | Inclusion criteria for this review are as follows: (1) primary studies using qualitative designs, (2) studies with participants who are individuals with chronic illness(es) and their caregivers, (3) studies focusing on transitional care between hospitals and homes delivered by nurses and other healthcare providers, (4) studies that included patients’ and their caregivers’ experiences and perceptions as a result of receiving transitional care, and (5) studies published as journal articles in English. | Full-text, peer-reviewed journals published in English were included. Eligible studies included adult Indigenous people (18 years or older) who received cancer care or services (including assessment, treatment, follow-up care and survivorship) and carers or family Int. J. Environ. Res. Public Health 2022, 19, 16947 3 of 13 members as the representatives of cancer survivors. There were no restrictions on cancer type, stage, or treatment. Only qualitative studies using interviews for data collection or secondary qualitative data were selected for the literature review. |  |  |  |
| Exclusion criteria | None reported | studies targeted solely on healthcare providers’ perspectives, studies focused on anything other than chronic illnesses with adult patients, those reporting patients’ experiences of anything other than hospital-to-home transitional care, and studies focused on care other than pre- or post-discharge care, bridging care, or care that otherwise does not meet the definition of “transitional care. | studies with (1) Indigenous people aged 18 years or less (those who were under 18 years but became adult cancer survivors were included), (2) healthcare professionals or related service providers or Aboriginal Liaison Officers (ALOs), (3) a focus on cancer screening only, (4) palliative care treatment only, (5) a focus on exploring knowledge about cancer only, or (6) reviews, book chapters, and abstracts published in conferences, editorial materials, correspondence, letters, case reports, newspaper materials, or dissertations |  |  |  |
| Initial number of studies | 5704 | 105 | 543 |  |  |  |
| Included number of studies | 18 | 7 | 23 |  |  |  |
| List included studies by author and publication year | Backman 2018, Backman 2019, Boye 2021, Du Goff 2016, Ekelund 2015, Gjevjon 2014, Grol 2020, Harvey 2018, Kanat 2021, Ljungholm 2021, MacInnes 2020, Mason 2016, Petrosyan 2020, Rustad 2016, Scholtz Mellum 2018, Summer Meranius 2016, Säfström 2018, Wong 2016 | Allen 2018, Backman 2018, Dolu 2021, Halding 2018, Reedy-Cooper 2020, Rijpkema 2021, Swan 2020 | Anderson 2021, Bell 2021, Green 2018, Lyford 2018, Marcusson-Rababi 2019, McGrath 2013a, McGrath 2013b, McGrath 2015, McMichael 2000, Meiklejohn 2017, Treloar 2013, Newman 2017, Reilly 2018, Ristevski 2020, Sariman 2022, Shahid 2009a, Shahid 2009b, Shahid 2010, Shahid 2011, Thompson 2011, Taylor 2020, Taylor 2021, Willis 2011 |  |  |  |
| Qualitative themes | (I) Patient experience, (II) Care over time | Barriers to transitional care: (I) Communication with multiple healthcare providers, (II) Self-management, (III) Psychological stress. Facilitators of transitional care: (I) Family caregivers support, (II) Nurse-provided patient-centered care | Key themes: (I) Communication, (II) Cultural safety, (III)Access to services |  |  |  |
