# Supplementary material for: Content validity of patient-reported measures evaluating experiences of the quality of transitions in healthcare settings—a scoping review
Source: BMC Health Serv Res. 2024 Jul 22;24:828. doi: 10.1186/s12913-024-11298-0 (PMC11265152; doi:10.1186/s12913-024-11298-0)
Supplement: Supplementary file 5 — Supplementary Material 5. [file 12913_2024_11298_MOESM5_ESM.docx]

# Instrument:

| Title: | Authors: | Year: |
| --- | --- | --- |
|  |  |  |
|  |  |  |
|  |  |  |

| **Box 2. Content validity** | | | | | | |
| --- | --- | --- | --- | --- | --- | --- |
| **2a. Asking patients about relevance** | | | | | | |
| *Design requirements* | | **very good** | **adequate** | **doubtful** | **inadequate** | **NA** |
|  | |  |  |  |  |  |
| 1 | Was an appropriate method used to ask patients whether each item is relevant for their experience with the condition? | Widely recognized or well justified method used | Only quantitative (survey) method(s) used or assumable that the method was appropriate but not clearly described | Not clear if patients were asked whether each item is relevant or doubtful whether the method was appropriate | Method used not appropriate or patients not asked about the relevance of all items |  |
| 2 | Was each item tested in an appropriate number of patients?  For qualitative studies  For quantitative (survey) studies | ≥7  ≥50 | 4-6  ≥30 | <4 or not clear  <30 or not clear |  |  |
| 3 | Were skilled group moderators/interviewers used? | Skilled group moderators/ interviewers used | Group moderators /interviewers had limited experience or were trained specifically for the study | Not clear if group moderators /interviewers were trained or group moderators /interviewers not trained and no experience |  | Not applicable |
| 4 | Were the group meetings or interviews based on an appropriate topic or interview guide? | Appropriate topic or interview guide | Assumable that the topic or interview guide was appropriate, but not clearly described | Not clear if a topic guide was used or doubtful if topic or interview guide was appropriate or no guide |  | Not applicable |
| 5 | Were the group meetings or interviews recorded and transcribed verbatim? | All group meetings or interviews were recorded and transcribed verbatim | Assumable that all group meetings or interviews were recorded and transcribed verbatim, but not clearly described | Not clear if all group meetings or interviews were recorded and transcribed verbatim or recordings not transcribed verbatim or only notes were made during the group meetings/ interviews | No recording and no notes | Not applicable |
| *Analyses* | |  |  |  |  |  |
|  |  |  |  |  |  |  |
| 6 | Was an appropriate approach used to analyse the data? | A widely recognized or well justified approach was used | Assumable that the approach was appropriate, but not clearly described | Not clear what approach was used or doubtful whether the approach was appropriate | Approach not appropriate |  |
| 7 | Were at least two researchers involved in the analysis? | At least two researchers involved in the analysis | Assumable that at least two researchers were involved in the analysis, but not clearly described | Not clear if two researchers were included in the analysis or only one researcher involved in the analysis |  |  |

| **2b Asking patients about comprehensiveness** | |  |  |  |  |  |
| --- | --- | --- | --- | --- | --- | --- |
| *Design requirements* | | **very good** | **adequate** | **doubtful** | **inadequate** | **NA** |
|  | |  |  |  |  |  |
| 8 | Was an appropriate method used for assessing the comprehensiveness of the PROM? | Widely recognized or well justified method used | Only quantitative (survey) method(s) used or assumable that the method was appropriate but not clearly described | Doubtful whether the method was appropriate | Method used not appropriate |  |
| 9 | Was each item tested in an appropriate number of patients?  For qualitative studies  For quantitative (survey) studies | ≥7  ≥50 | 4-6  ≥30 | <4 or not clear  <30 or not clear |  |  |
| 10 | Were skilled group moderators/interviewers used? | Skilled group moderators/ interviewers used | Group moderators /interviewers had limited experience or were trained specifically for the study | Not clear if group moderators /interviewers were trained or group moderators /interviewers not trained and no experience |  | Not applicable |
| 11 | Were the group meetings or interviews based on an appropriate topic or interview guide? | Appropriate topic or interview guide | Assumable that the topic or interview guide was appropriate, but not clearly described | Not clear if a topic guide was used or doubtful if topic or interview guide was appropriate or no guide |  | Not applicable |
| 12 | Were the group meetings or interviews recorded and transcribed verbatim? | All group meetings or interviews were recorded and transcribed verbatim | Assumable that all group meetings or interviews were recorded and transcribed verbatim, but not clearly described | Not clear if all group meetings or interviews were recorded and transcribed verbatim or recordings not transcribed verbatim or only notes were made during the group meetings/ interviews | No recording and no notes | Not applicable |
| *Analyses* | |  |  |  |  |  |
|  | |  |  |  |  |  |
| 13 | Was an appropriate approach used to analyse the data? | A widely recognized or well justified approach was used | Assumable that the approach was appropriate, but not clearly described | Not clear what approach was used or doubtful whether the approach was appropriate | Approach not appropriate |  |
| 14 | Were at least two researchers involved in the analysis? | At least two researchers involved in the analysis | Assumable that at least two researchers were involved in the analysis, but not clearly described | Not clear if two researchers were included in the analysis or only one researcher involved in the analysis |  |  |

| **2c Asking patients about comprehensibility** | | | | | | |
| --- | --- | --- | --- | --- | --- | --- |
| *Design requirements* | | **very good** | **adequate** | **doubtful** | **inadequate** | **NA** |
|  | |  |  |  |  |  |
| 15 | Was an appropriate qualitative method used for assessing the comprehensibility of the PROM instructions, items, response options, and recall period? | Widely recognized or well justified qualitative method used | Assumable that the method was appropriate but not clearly described | Only quantitative (survey) method(s) used or doubtful whether the method was appropriate or not clear if patients were asked about the comprehensibility of the items, response options or recall period or patients not asked about the comprehensibility of the PROM instructions | Method used not appropriate or patients not asked about the comprehensibility of the items, response options, or recall period |  |
| 16 | Was each item tested in an appropriate number of patients?  For qualitative studies  For quantitative (survey) studies | ≥7  ≥50 | 4-6  ≥30 | <4 or not clear  <30 or not clear |  |  |
| 17 | Were skilled group moderators/interviewers used? | Skilled group moderators/ interviewers used | Group moderators /interviewers had limited experience or were trained specifically for the study | Not clear if group moderators /interviewers were trained or group moderators /interviewers not trained and no experience |  |  |
| 18 | Were the group meetings or interviews based on an appropriate topic or interview guide? | Appropriate topic or interview guide | Assumable that the topic or interview guide was appropriate, but not clearly described | Not clear if a topic guide was used or doubtful if topic or interview guide was appropriate or no guide |  | Not applicable |
| 19 | Were the group meetings or interviews recorded and transcribed verbatim? | All group meetings or interviews were recorded and transcribed verbatim | Assumable that all group meetings or interviews were recorded and transcribed verbatim, but not clearly described | Not clear if all group meetings or interviews were recorded and transcribed verbatim or recordings not transcribed verbatim or only notes were made during the group meetings/ interviews | No recording and no notes | Not applicable |
| *Analyses* | |  |  |  |  |  |
|  | |  |  |  |  |  |
| 20 | Was an appropriate approach used to analyse the data? | A widely recognized or well justified approach was used | Assumable that the approach was appropriate, but not clearly described | Not clear what approach was used or doubtful whether the approach was appropriate | Approach not appropriate |  |
| 21 | Were at least two researchers involved in the analysis? | At least two researchers involved in the analysis | Assumable that at least two researchers were involved in the analysis, but not clearly described | Not clear if two researchers were included in the analysis or only one researcher involved in the analysis |  |  |
