# Supplementary material for: Content validity of patient-reported measures evaluating experiences of the quality of transitions in healthcare settings—a scoping review
Source: BMC Health Serv Res. 2024 Jul 22;24:828. doi: 10.1186/s12913-024-11298-0 (PMC11265152; doi:10.1186/s12913-024-11298-0)
Supplement: Supplementary file 6 — Supplementary Material 6. [file 12913_2024_11298_MOESM6_ESM.docx]

| Patient-Reported Experience Measure (PREM) | Reason for exclusion | Articles | Original development and/or evaluation articles |
| --- | --- | --- | --- |
| Abbreviation and full name |  | First author and year* | First author and year* |
| Care Continuity Instrument | < 5 domains represented, items not available in question format | Bull 2000 |  |
| PACT-M Partners at Care Transitions Measure | < 5 domains represented | Liu 2021, Oikonomou 2020, Yoshimura 2022 |  |
| PCCCT Patient-centered Coordination by a Care Team questionnaire | < 5 domains represented | Ramond-Roquin 2019 |  |
| PREMIUM-ACC The Patient-Reported Experience Measure for Improving qUality of care in Mental health | < 5 domains represented | Boyer 2023 |  |
| PREPARED and B-PREPARED Prescriptions, Ready to re-enter community, Education, Placement, Assurance of safety, Realistic expectations, Empowerment, Directed to appropriate services questionnaire | < 5 domains represented, wrong phenomenon of interest | Hwang 2019, Andrew 2018, Yoshimura 2022 | Grimmer 2001 |
| IEXPAC Chronic Patient Experience Evaluation Instrument | < 5 domains represented | Mira 2016, Guilabert 2021 |  |
| QUOTE and QUOTE-IBD Quality of care through the patient’s eyes | < 5 domains represented | Soares 2015, Berbee 2009, McMurray 2016, Bortoli 2014 | Sixma 1998, van der Linde 2007 |
| OPTION Opportunity for Treatment In Oncology questionnaire | < 5 domains represented, items not available in question format | Rucci 2018, Bravi 2018 |  |
| NHHSUQ National Health and Health Services Use Questionnaire | Items not available in question format | Bentler 2014, Bentler 2014, Bentler 2014 |  |
| CAHPS 2.0 (Core) Consumer Assessment of Healthcare Providers and Systems | Wrong phenomenon of interest | Berbee 2009, Black 2021, Fiscella 2011, Quinn 2017, Wells 2020, Sequist 2012, O’Malley 2009, Mollica 2021 | Hargraves 2003, Platonova 2016, Dyer 2012, Hays 2013 |
| CSC the Coordination of Specialty Care – Patient | Wrong phenomenon of interest | Vimalananda 2023 |  |
| MHSSS Mental Health Service Satisfaction Scale | Wrong phenomenon of interest | Fernandes 2020 | Mayston 2017 |
| MISS-21 Medical Interview Satisfaction Scale | Wrong phenomenon of interest | Berbee 2009 | Meakin 2002, Slade 2010 |
| MQOC Menninger Quality of Care | Wrong phenomenon of interest | Fernandes 2020 | Madan 2014 |
| MRPS MedRisk Instrument for measuring Patient Satisfaction with physical therapy care | Wrong phenomenon of interest | McMurray 2016 | Beattie 2005, Hush 2013 |
| Neurorehabilitation Experience Questionnaire | Wrong phenomenon of interest | McMurray 2016 | Kneebone 2012 |
| Press Ganey Medical Practice Survey – 8 domain | Wrong phenomenon of interest | Black 2021 | Day 2013 |
| Press Ganey Patient Satisfaction Survey | Wrong phenomenon of interest | Black 2021 | Carillo 2014 |
| PRP American Diabetes Association-National Committee for Quality Assurance Provider Recognition Program Survey | Wrong phenomenon of interest | Berbee 2009 | Montori 2002 |
| 4PAS 4-Point ordinal Alliance Self-report | Wrong phenomenon of interest | Fernandes 2020 | Misdrahi 2009 |
| ACES Ambulatory Care Experiences Survey | Wrong phenomenon of interest | Quinn 2017 | Safran 2006 |
| CABHS Consumer Assessment of Behavioral Health Services | Wrong phenomenon of interest | Fernandes 2020 | Eisen 1999, Eisen 2001 |
| CAHPS-PCMH | Wrong phenomenon of interest | Black 2021, Quinn 2017 | Nelson 2014, Hayes 2014 |
| CANHELP and CANHELP-lite Canadian Health Care Evaluation Project Questionnaire | Wrong phenomenon of interest | Quinn 2017, den Herder-van der Eerden 2018 | Heyland 2010, Heyland 2013 |
| CASC Comprehensive Assessment of Satisfaction with Care | Wrong phenomenon of interest | Berbee 2009 | Brédart 1999 |
| CCCQ-P Camden Content of Care Questionnaire – Patient version | Wrong phenomenon of interest | Fernandes 2020, Collett 2019 | Lloyd-Evans 2010 |
| CCEQ Chronic Cancer Experiences Questionnaire | Wrong phenomenon of interest | Boele 2019 | Harley 2019 |
| CEO-MHS Consumer Evaluation of Mental Health Services | Wrong phenomenon of interest | Fernandes 2020 | Oades 2011, Rose 2011 |
| CONNECT A Measure of Continuity of Care in Mental Health Services | Wrong phenomenon of interest | Fernandes 2020, Weaver 2017 | Ware 2003 |
| CPOSS Charleston Psychiatric Outpatient Satisfaction Scale | Wrong phenomenon of interest | Fernandes 2020 | Pellegrin 2001 |
| CSQ Client Satisfaction Questionnaire | Wrong phenomenon of interest | Berbee 2009, Fernandes 2020 | Attkisson 1996, Larsen 1979, Koch 1995 |
| CSQ Consultation Satisfaction Questionnaire | Wrong phenomenon of interest | Fernandes 2020 | Baker 1990 |
| CSQ Consumer Satisfaction Questionnaire | Wrong phenomenon of interest | Fernandes 2020 | Brunero 2009 |
| CUPSS Clinically Useful Patient Satisfaction Scale | Wrong phenomenon of interest | Fernandes 2020 | Zimmerman 2017 |
| CWF Commonwealth Fund International Health Policy Survey | Wrong phenomenon of interest | Hincapie 2016, Burgers 2010 |  |
| DTSQ The Diabetes Treatment Satisfaction Questionnaire | Wrong phenomenon of interest | Berbee 2009 | Bradley 2007 |
| ECS Evaluation of Client Services | Wrong phenomenon of interest | Fernandes 2020 | Berghofer 2011 |
| EDITS Erectile Dysfunction Inventory of Treatment Satisfaction | Wrong phenomenon of interest | Berbee 2009 | Althof 1999 |
| EORTC-QLQ- SAT32 European Organisation for Research and Treatment of Cancer cancer inpatient satisfaction questionnaire | Wrong phenomenon of interest | Fillion 2012, Fillion 2009 | Brédart 2004 |
| ERS Evaluation Rinking Scale | Wrong phenomenon of interest | Berbee 2009 | Pascoe 1983, Koch 1995 |
| EUROPEP instrument for measuring patient evaluation of general practice care in Europe | Wrong phenomenon of interest | Berbee 2009, Quuinn 2017 | Wensing 2000 |
| GSQ General Satisfaction Questionnaire | Wrong phenomenon of interest | Berbee 2009 | Huxley 1992 |
| Health Care System Hassles Scale | Wrong phenomenon of interest | Noël 2020 | Parchman 2005 |
| Healthcare Climate Questionnaire | Wrong phenomenon of interest | Black 2021 | Ciechanowski 2006 |
| HPIQ Health Promotion Intervention Questionnaire | Wrong phenomenon of interest | Fernandes 2020 | Svedberg 2008, Svedberg 2007 |
| ICS Inpatient Consumer Survey | Wrong phenomenon of interest | Fernandes 2020 | Ortiz 2012 |
| IESQ Inpatient Evaluation of Service Questionnaire | Wrong phenomenon of interest | Fernandes 2020 | Meehan 2002 |
| Inpatient Psychiatric Questionnaire | Wrong phenomenon of interest | Fernandes 2020 | Kolb 2000 |
| ISQ Inpatient Satisfaction Questionnaire | Wrong phenomenon of interest | Fernandes 2020 | Shiva 2011 |
| ITSQ Insulin Treatment Satisfaction Questionnaire | Wrong phenomenon of interest | Berbee 2009 | Anderson 2004 |
| KY-CSI Kentucky Consumer Satisfaction Instrument | Wrong phenomenon of interest | Fernandes 2020 | Howard 2001 |
| Maternal Satisfaction Questionnaire | Wrong phenomenon of interest | Perriman 2016 | Fereday 2009 |
| OPEQ Outpatient Experience Questionnaire | Wrong phenomenon of interest | McMurray 2016 | Normann 2012 |
| PACE SQ Program for All-Inclusive Care of the Elderly Satisfaction Survey | Wrong phenomenon of interest | Berbee 2009 | Atherly 2004 |
| Patient Provider Relationship Questionnaire | Wrong phenomenon of interest | Black 2021 | Smith 2009 |
| Patient Satisfaction Survey | Wrong phenomenon of interest | Fernandes 2020 | Parker 1996 |
| PCAT Primary Care Assessement Tool | Wrong phenomenon of interest | Fillion 2012, Quinn 2017 | Shi 2001 |
| PCQ-H Primary Care Quality – Homeless | Wrong phenomenon of interest | Fernandes 2020 | Kertesz 2014 |
| PCQCCQ-S | Wrong phenomenon of interest | Volakakis 2024 | Doubova 2021 |
| PCSQ Psychiatric Care Questionnaire | Wrong phenomenon of interest | Fernandes 2020 | Barker 1999, Barker 1996 |
| PEACS Patients’ Experiences Across Health Care Sectors | Wrong phenomenon of interest | Noest 2014 |  |
| PEC-5 Patient Evaluation of Care - 5 | Wrong phenomenon of interest | Fernandes 2020 | Blais 2002 |
| PEQ Patient experience questionnaire | Wrong phenomenon of interest | Fernandes 2020, McMurray 2016 | Mavaddat 2009, Slade 2010, Steine 2001 |
| Physiotherapy Outpatient Survey | Wrong phenomenon of interest | McMurray 2016 | French 2010 |
| PIPEQ-OS Psychiatric Inpatient Patient Experience Questionnaire on-site version | Wrong phenomenon of interest | Fernandes 2020 | Bjertnæs 2015 |
| PoC Perception of care | Wrong phenomenon of interest | Fernandes 2020 | Eisen 2002 |
| POPEQ Psychiatric Out-Patient Experiences Questionnaire | Wrong phenomenon of interest | Fernandes 2020 | Garrat 2006, Olsen 2010 |
| PPE-15 Picker Patient Experience Questionnaire | Wrong phenomenon of interest | Berbee 2009, Fernandes 2020, McMurray 2016, Weinberg 2007 | Jenkinson 2002, Slade 2010, Jenkinson 2003 |
| PSH Satisfaction and Participatory Style Questionnaire | Wrong phenomenon of interest | Berbee 2009 | Lubeck 2000 |
| PSHCPS Patient Satisfaction with Health Care Provider Scale | Wrong phenomenon of interest | Berbee 2009 | Marsh 1999 |
| PSI Patient Satisfaction Interview | Wrong phenomenon of interest | Berbee 2009 | Corrigan 1993 |
| PSQ-18 Patient Satisfaction Questionnaire | Wrong phenomenon of interest | Berbee 2009, Black 2021, McMurray 2016, Alsayali 2019 | RAND Health 1994, Zulman 2014, Roush 2007 |
| PTOPS Physical Therapy Outpatient Satisfaction Survey | Wrong phenomenon of interest | McMurray 2016 | Roush 2007, Vanti 2013 |
| PTPSQ Physical Therapy Patient Satisfaction Questionnaire | Wrong phenomenon of interest | McMurray 2016 | Duijin 2008, Slade 2010, Goldstein 2000 |
| QPC-IP and QPC-OP Quality in Psychiatric Care - Inpatient and Outpatient | Wrong phenomenon of interest | Fernandes 2020 | Schröder 2007, Schröder 2010, Schröder 2010 |
| QPCC Patient- Centered Quality of Cancer Care Questionnaire | Wrong phenomenon of interest | Volakakis 2024 | Tzelepis 2015 |
| Rehabilitation Patient Experiences Questionnaire | Wrong phenomenon of interest | McMurray 2016 | Grotle 2009 |
| SAT-16 Patient satisfaction in rehabilitative therapy | Wrong phenomenon of interest | Berbee 2009, McMurray 2016 | Franchignoni 1998, Ottonello 2012, Koch 1995 |
| Satisfaction with antenatal care, intrapartum care, and postnatal care | Wrong phenomenon of interest | Perriman 2016 | Waldenström 2003 |
| SAT-P Satisfaction Profile | Wrong phenomenon of interest | Berbee 2009 | Majani 1999 |
| SEQUenCE Service user Quality of Care | Wrong phenomenon of interest | Fernandes 2020 | Hester 2015 |
| SERVQUAL Model of service quality gaps | Wrong phenomenon of interest | McMurray 2016 | Curry 2002 |
| SHC Satisfaction with Health Care | Wrong phenomenon of interest | Berbee 2009 | Hall 1999 |
| SI-MH Satisfaction Index - Mental Health | Wrong phenomenon of interest | Fernandes 2020 | Nabati 1998 |
| SMHC Satisfaction with Mental Health Care | Wrong phenomenon of interest | Fernandes 2020 | Slater 1982 |
| SPRI Self-Rating Patient Questionnaire | Wrong phenomenon of interest | Fernandes 2020 | Hansson 1995 |
| SSS-30 Service Satisfaction Scale | Wrong phenomenon of interest | Berbee 2009 | Attkinson 1994 |
| STAR-P Scale To Assess the therapeutic Relationship | Wrong phenomenon of interest | Fernandes 2020 | McGuire 2007 |
| TPS Trust in Physician Scale | Wrong phenomenon of interest | Fernandes 2020 | Aloba 2014, Anderson 1990 |
| TSQM Treatment Satisfaction Questionnaire for Medication | Wrong phenomenon of interest | Berbee 2009 | Atkinson 2004 |
| VOICE Views On Inpatient Care | Wrong phenomenon of interest | Fernandes 2020 | Evans 2012 |
| VSSS Verona Service Satisfaction Scale | Wrong phenomenon of interest | Berbee 2009, Fernandes 2020, Kessing 2006 | Ruggeri 1993, Ruggeri 2000 |

* For full reference list contact first author
