# Supplementary material for: Content validity of patient-reported measures evaluating experiences of the quality of transitions in healthcare settings—a scoping review
Source: BMC Health Serv Res. 2024 Jul 22;24:828. doi: 10.1186/s12913-024-11298-0 (PMC11265152; doi:10.1186/s12913-024-11298-0)
Supplement: Supplementary file 7 — Supplementary Material 7. [file 12913_2024_11298_MOESM7_ESM.pdf]

27 reports (1–27) for objective 1:

1. Allen J, Hutchinson AM, Brown R, Livingston PM. User experience and care for older people transitioning from hospital to home: Patients' and carers' perspectives. Health Expect Int J Public Particip Health Care Health Policy. 2018;21(2):518–27.

2. Instituto Nacional de Cancerología E.S.E, Arias Rojas M, García-Vivar C, Universidad de Navarra. The transition of palliative care from the hospital to the home: a narrative review of experiences of patients and family caretakers. Investig Educ En Enferm [Internet]. 15. oktober 2015 [henvist 17. november 2022];33(3). Tilgængelig hos: https://revistas.udea.edu.co/index.php/iee/article/view/24457

3. Asif M, Cadel L, Kuluski K, Everall AC, Guilcher SJT. Patient and caregiver experiences on care transitions for adults with a hip fracture: a scoping review. Disabil Rehabil. 19. november 2020;42(24):3549–58.

4. Beattie M, Shepherd A, Howieson B. Do the Institute of Medicine's (IOM's) dimensions of quality capture the current meaning of quality in health care? – An integrative review. J Res Nurs. juni 2013;18(4):288–304.

5. Boye LK, Mogensen CB, Mechlenborg T, Waldorff FB, Andersen PT. Older multimorbid patients' experiences on integration of services: a systematic review. BMC Health Serv Res. december 2019;19(1):795.

6. Carpenter JG. Hospital Palliative Care Teams and Post-Acute Care in Nursing Facilities: An Integrative Review. Res Gerontol Nurs. januar 2017;10(1):25–34.

7. Davidson L, Scott J, Forster N. Patient experiences of integrated care within the United Kingdom: A systematic review. Int J Care Coord. juni 2021;24(2):39–56.

8. De Regge M, De Pourcq K, Meijboom B, Trybou J, Mortier E, Eeckloo K. The role of hospitals in bridging the care continuum: a systematic review of coordination of care and follow-up for adults with chronic conditions. BMC Health Serv Res. december 2017;17(1):550.

9. Foglino S, Bravi F, Carretta E, Fantini MP, Dobrow MJ, Brown AD. The relationship between integrated care and cancer patient experience: A scoping review of the evidence. Health Policy. januar 2016;120(1):55–63.

10. Hestevik CH, Molin M, Debesay J, Bergland A, Bye A. Older persons' experiences of adapting to daily life at home after hospital discharge: a qualitative metasummary. BMC Health Serv Res. december 2019;19(1):224.

11. Hohmann NS, McDaniel CC, Mason SW, Cheung WY, Williams MS, Salvador C, m.fl. Patient perspectives on primary care and oncology care coordination in the context of multiple chronic conditions: A systematic review. Res Soc Adm Pharm. august 2020;16(8):1003–16.

12. Lawless MT, Marshall A, Mittinty MM, Harvey G. What does integrated care mean from an older person's perspective? A scoping review. BMJ Open. januar 2020;10(1):e035157.

13. Mayo SJ, Ajaj R, Drury A. Survivors' preferences for the organization and delivery of supportive care after treatment: An integrative review. Eur J Oncol Nurs. oktober 2021;54:102040.

14. McMurray J, McNeil H, Lafortune C, Black S, Prorok J, Stolee P. Measuring Patients' Experience of Rehabilitation Services Across the Care Continuum. Part II: Key Dimensions. Arch Phys Med Rehabil. 2016;97(1):121–30.

15. Oishi A, Murtagh FE. The challenges of uncertainty and interprofessional collaboration in palliative care for non-cancer patients in the community: A systematic review of views from patients, carers and health-care professionals. Palliat Med. oktober 2014;28(9):1081–98.

16. Piccenna L, Lannin NA, Gruen R, Pattuwage L, Bragge P. The experience of discharge for patients with an acquired brain injury from the inpatient to the community setting: A qualitative review. Brain Inj. 23. februar 2016;30(3):241–51.

17. Sampson R, Cooper J, Barbour R, Polson R, Wilson P. Patients' perspectives on the medical primary–secondary care interface: systematic review and synthesis of qualitative research. BMJ Open. oktober 2015;5(10):e008708.

18. Scholl I, Zill JM, Härter M, Dirmaier J. An Integrative Model of Patient-Centeredness – A Systematic Review and Concept Analysis. Wu WCH, redaktør. PLoS ONE. 17. september 2014;9(9):e107828.

19. Segan JD, Briggs AM, Chou L, Connelly KL, Seneviwickrama M, Sullivan K, m.fl. Patient-perceived health service needs in inflammatory arthritis: A systematic scoping review. Semin Arthritis Rheum. juni 2018;47(6):765–77.

20. Sibounheuang P, Olson PS, Kittiboonyakun P. Patients' and healthcare providers' perspectives on diabetes management: A systematic review of qualitative studies. Res Soc Adm Pharm. juli 2020;16(7):854–74.

21. Staniszewska S, Boardman F, Gunn L, Roberts J, Clay D, Seers K, m.fl. The Warwick Patient Experiences Framework: patient-based evidence in clinical guidelines. Int J Qual Health Care. 1. april 2014;26(2):151–7.

22. van Servellen G, Fongwa M, Mockus D'Errico E. Continuity of care and quality care outcomes for people experiencing chronic conditions: A literature review. Nurs Health Sci. september 2006;8(3):185–95.

23. Waibel S, Henao D, Aller MB, Vargas I, Vazquez ML. What do we know about patients' perceptions of continuity of care? A meta-synthesis of qualitative studies. Int J Qual Health Care. 1. februar 2012;24(1):39–48.

24. Youssef A, Chaudhary ZK, Wiljer D, Mylopoulos M, Sockalingam S. Mapping Evidence of Patients' Experiences in Integrated Care: A Scoping Review. Gen Hosp Psychiatry. november 2019;61:1–9.

25. Djukanovic I, Hellström A, Wolke A, Schildmeijer K. The meaning of continuity of care from the perspective of older people with complex care needs–A scoping review. Geriatr Nur (Lond). januar 2024;55:354–61.

26. Joo JY, Liu MF. The Experience of Chronic Illness Transitional Care: A Qualitative Systematic Review. Clin Nurs Res. februar 2022;31(2):163–73.

27. Sanjida S, Garvey G, Ward J, Bainbridge R, Shakeshaft A, Hadikusumo S, m.fl. Indigenous Australians' Experiences of Cancer Care: A Narrative Literature Review. Int J Environ Res Public Health. 16. december 2022;19(24):16947.

163 reports for objective 2: 77 observational/survey studies: (28–104); 12 reviews of instruments: (105–116); 73 development of instrument and evaluation of measurement properties studies (117–189)

28. Ade A, Debroucker F, Delporte L, De Monclin C, Fayet E, Legendre P, m.fl. Chronic patients' satisfaction and priorities regarding medical care, information and services and quality of life: a French online patient community survey. BMC Health Serv Res. 2020;20(1):511.

29. Aller MB, Vargas I, Waibel S, Coderch-Lassaletta J, Sánchez-Pérez I, Llopart JR, m.fl. Factors associated to experienced continuity of care between primary and outpatient secondary care in the Catalan public healthcare system. Gac Sanit. maj 2013;27(3):207–13.

30. Alsayali MM, Al-Sahafi A, Mandoura N, Usman Shah HB, Abdul Rashid OA, AlSharif K, m.fl. Patients' Satisfaction after Primary Health Care Centers' Integration with Ministry of Health Hospitals, Jeddah. J Epidemiol Glob Health. 2019;135.

31. Andrew NE, Busingye D, Lannin NA, Kilkenny MF, Cadilhac DA. The Quality of Discharge Care Planning in Acute Stroke Care: Influencing Factors and Association with Postdischarge Outcomes. J Stroke Cerebrovasc Dis. marts 2018;27(3):583–90.

32. Arnold C, Hennrich P, Wensing M. Patient-reported continuity of care and the association with patient experience of cardiovascular prevention: an observational study in Germany. BMC Prim Care. december 2022;23(1):176.

33. Auerbach AD, Kripalani S, Vasilevskis EE, Sehgal N, Lindenauer PK, Metlay JP, m.fl. Preventability and Causes of Readmissions in a National Cohort of General Medicine Patients. JAMA Intern Med. 1. april 2016;176(4):484.

34. Barimani M, Oxelmark L, Johansson SE, Hylander I. Support and continuity during the first 2 weeks postpartum. Scand J Caring Sci. 2015;29(3):409–17.

35. Bentler SE, Morgan RO, Virnig BA, Wolinsky FD. The Association of Longitudinal and Interpersonal Continuity of Care with Emergency Department Use, Hospitalization, and Mortality among Medicare Beneficiaries. Hernandez-Boussard T, redaktør. PLoS ONE. 22. december 2014;9(12):e115088.

36. Benzer JK, Singer SJ, Mohr DC, McIntosh N, Meterko M, Vimalananda VG, m.fl. Survey of Patient-Centered Coordination of Care for Diabetes with Cardiovascular and Mental Health Comorbidities in the Department of Veterans Affairs. J Gen Intern Med. maj 2019;34(S1):43–9.

37. Boele F, Harley C, Pini S, Kenyon L, Daffu-O'Reilly A, Velikova G. Cancer as a chronic illness: support needs and experiences. BMJ Support Palliat Care. 19. september 2019;bmjspcare-2019-001882.

38. Bortoli A, Daperno M, Kohn A, Politi P, Marconi S, Monterubbianesi R, m.fl. Patient and physician views on the quality of care in inflammatory bowel disease: Results from SOLUTION-1, a prospective IG-IBD study. J Crohns Colitis. december 2014;8(12):1642–52.

39. Bower P, Reeves D, Sutton M, Lovell K, Blakemore A, Hann M, m.fl. Improving care for older people with long-term conditions and social care needs in Salford: the CLASSIC mixed-methods study, including RCT. Health Serv Deliv Res. august 2018;6(31):1–188.

40. Brant J, Blaseg K, Aders K, Oliver D, Gray E, Dudley W. Navigating the Transition From Cancer Care to Primary Care: Assistance of a Survivorship Care Plan. Oncol Nurs Forum. 1. november 2016;43(6):710–9.

41. Bravi F, Ruscio ED, Frassoldati A, Cavallesco GN, Valpiani G, Ferrozzi A, m.fl. Patient and Health Care Professional Perspectives: A Case Study of the Lung Cancer Integrated Care Pathway. Int J Integr Care. 31. oktober 2018;18(4):7.

42. Brennan ME, Butow P, Spillane AJ, Boyle F. Patient-reported quality of life, unmet needs and care coordination outcomes: Moving toward targeted breast cancer survivorship care planning: Quality of life in early breast cancer. Asia Pac J Clin Oncol. juni 2016;12(2):e323–31.

43. Breton M, Haggerty J, Roberge D, Freeman GK. Management continuity in local health networks. Int J Integr Care. 2012;12(101214424):e14.

44. Burgers JS, Voerman GE, Grol R, Faber MJ, Schneider EC. Quality and Coordination of Care for Patients With Multiple Conditions: Results From an International Survey of Patient Experience. Eval Health Prof. september 2010;33(3):343–64.

45. Carneiro C, Ellis J, Singh S, Cheung MC. Patient and Primary Care Practitioner Confidence in and Perceptions of Cancer Treatment Transition and the Shared Care Model of Cancer Care. J Oncol Navig Surviv. 2016;7(8):18–24.

46. Carryer J, Doolan-Noble F, Gauld R, Budge C. New Zealand patients' perceptions of chronic care delivery. J Integr Care. 14. april 2014;22(2):71–80.

47. Chen CC, Cheng SH. Reexamining the association of care continuity and health care outcomes. Am J Manag Care. 1. august 2023;29(8):e242–9.

48. Collett GK, Durcinoska I, Rankin NM, Blinman P, Barnes DJ, Anderiesz C, m.fl. Patients' experience of lung cancer care coordination: a quantitative exploration. Support Care Cancer. februar 2019;27(2):485–93.

49. Cramm JM, Nieboer AP. High-quality chronic care delivery improves experiences of chronically ill patients receiving care. Int J Qual Health Care. 1. december 2013;25(6):689–95.

50. Dean JE, Hutchinson A, Escoto KH, Lawson R. Using a multi-method, user centred, prospective hazard analysis to assess care quality and patient safety in a care pathway. BMC Health Serv Res. december 2007;7(1):89.

51. den Herder-van der Eerden M, Ebenau A, Payne S, Preston N, Radbruch L, Linge-Dahl L, m.fl. Integrated palliative care networks from the perspectives of patients: A cross-sectional explorative study in five European countries. Palliat Med. 2018;32(6):1103–13.

52. Durcinoska I, Young JM, Solomon MJ. Patterns and predictors of colorectal cancer care coordination: A population-based survey of Australian patients: Predictors of Cancer Care Coordination. Cancer. 15. januar 2017;123(2):319–26.

53. Erlang AS, Schjødt K, Linde JKS, Jensen AL. An observational study of older patients' experiences of involvement in discharge planning. Geriatr Nur (Lond). juli 2021;42(4):855–62.

54. Fillion L, De Serres M, Cook S, Goupil RL, Bairati I, Doll R. Professional Patient Navigation in Head and Neck Cancer. Semin Oncol Nurs. august 2009;25(3):212–21.

55. Fitch MI, Nicoll I, Lockwood G. Positive Cancer Experiences: Perspectives From Cancer Survivors. J Patient Exp. 2020;7(6):1501–8.

56. Fryer AK, Friedberg MW, Thompson RW, Singer SJ. Achieving care integration from the patients' perspective: Results from a care management program. Healthcare. marts 2016;4(1):36–44.

57. Gomez-Cano M, Lyratzopoulos G, Abel GA. Patient Experience Drivers of Overall Satisfaction With Care in Cancer Patients: Evidence From Responders to the English Cancer Patient Experience Survey. J Patient Exp. 2020;7(5):758–65.

58. Gottberg K, Einarsson U, Ytterberg C, Fredrikson S, von Koch L, Holmqvist LW. Use of health care services and satisfaction with care in people with multiple sclerosis in Stockholm County: a population-based study. Mult Scler Houndmills Basingstoke Engl. 2008;14(7):962–71.

59. Guilabert M, Martínez-García A, Sala-González M, Solas O, Mira JJ. Results of a Patient Reported Experience Measure (PREM) to measure the rare disease patients and caregivers experience: a Spanish cross-sectional study. Orphanet J Rare Dis. december 2021;16(1):67.

60. Hansen AH, Lian OS. How do women with chronic fatigue syndrome/myalgic encephalomyelitis rate quality and coordination of healthcare services? A cross-sectional study. BMJ Open. 2016;6(4):e010277.

61. Hincapie AL, Slack M, Malone DC, MacKinnon NJ, Warholak TL. Relationship Between Patients' Perceptions of Care Quality and Health Care Errors in 11 Countries: A Secondary Data Analysis. Qual Manag Health Care. januar 2016;25(1):13–21.

62. Hod R, Maimon O, Zimlichman E. Does Care Transition Matter? Exploring the Newly Published HCAHPS Measure. Am J Med Qual. september 2020;35(5):380–7.

63. Hopstaken JS, van Dalen D, van der Kolk BM, van Geenen EJM, Hermans JJ, Gootjes EC, m.fl. Continuity of care experienced by patients in a multi-institutional pancreatic care network: a pilot study. BMC Health Serv Res. 2021;21(1):1–9.

64. Huber DL, McClelland E. Patient preferences and discharge planning transitions. J Prof Nurs Off J Am Assoc Coll Nurs. 2003;19(4):204–10.

65. Ireson CL, Scutchfield FD, Slavova S, Steltenkamp CL. Bridgingf the care continuum: Patient information needs for specialist referrals. BMC Health Serv Res. 2009;9((Ireson, Scutchfield) College of Public Health, University of Kentucky, Lexington, KY, United States(Slavova) Office of Institutional Research, University of Kentucky, Lexington, KY, United States(Steltenkamp) College of Medicine, University of Kentucky,):163.

66. Karam M, Lambert AS, Macq J. Patients' perceptions of continuity of care across primary care level and emergency departments in Belgium: cross-sectional survey. BMJ Open. december 2019;9(12):e033188.

67. Kessing LV, Hansen HV, Ruggeri M, Bech P. Satisfaction with treatment among patients with depressive and bipolar disorders. Soc Psychiatry Psychiatr Epidemiol. februar 2006;41(2):148–55.

68. Khanbhai M, Warren L, Symons J, Flott K, Harrison-White S, Manton D, m.fl. Using natural language processing to understand, facilitate and maintain continuity in patient experience across transitions of care. Int J Med Inf. 2022;157(ct4, 9711057):104642.

69. Kjaer ML, Mainz J, Sorensen LT, Karlsmark T, Gottrup F. Venous leg ulcer patient priorities and quality of care: results of a survey. Ostomy Wound Manage. 2004;50(1):48–55.

70. Kollen BJ, Groenier KH, Berendsen AJ. Patients' experiences with continuum of care across hospitals. A multilevel analysis of Consumer Quality Index Continuum of Care. Patient Educ Couns. maj 2011;83(2):269–72.

71. Lafferty J, Rankin F, Duffy C, Kearney P, Doherty E, McMenamin M, m.fl. Continuity of care for women with breast cancer: A survey of the views and experiences of patients, carers and health care professionals. Eur J Oncol Nurs. 2011;15(5):419–27.

72. Lindegaard BR, Qvist P. Analysis of patient experiences regarding continuity of care. Nord Nurs Res Nord Sygeplejeforskning. 2012;2(4):307–13.

73. Liu LM, Zhuansun MY, Xu TY, Qian YM, Zhang HQ, Zhang QH, m.fl. Measuring the quality of transitional care based on elderly patients' experiences with the partners at care transitions measure: a cross-sectional survey. BMC Nurs. 14. marts 2024;23(1):172.

74. Lo C, Teede H, Fulcher G, Gallagher M, Kerr PG, Ranasinha S, m.fl. Gaps and barriers in health-care provision for co-morbid diabetes and chronic kidney disease: a cross-sectional study. BMC Nephrol. 2017;18(1):80.

75. Lundstrom LH, Johnsen AT, Ross L, Petersen MA, Groenvold M. Cross-sectorial cooperation and supportive care in general practice: cancer patients' experiences. Fam Pract. 2011;28(5):532–40.

76. Martinez KA, Snyder CF, Malin JL, Dy SM. Patient-reported quality of care and pain severity in cancer. Palliat Support Care. 2015;13(4):875–84.

77. Mastellos N, Gunn L, Harris M, Majeed A, Car J, Pappas Y. Assessing patients' experience of integrated care: a survey of patient views in the North West London Integrated Care Pilot. Int J Integr Care. 2014;14(101214424):e015.

78. Mendes FRP, Gemito MLGP, Caldeira E do C, Serra I da C, Casas-Novas MV. Continuity of care from the perspective of users. Cienc Saude Coletiva. 2017;22(3):841–53.

79. Mohr DC, Benzer JK, Vimalananda VG, Singer SJ, Meterko M, McIntosh N, m.fl. Organizational Coordination and Patient Experiences of Specialty Care Integration. J Gen Intern Med. maj 2019;34(S1):30–6.

80. Mollica MA, Buckenmaier SS, Halpern MT, McNeel TS, Weaver SJ, Doose M, m.fl. Perceptions of care coordination among older adult cancer survivors: A SEER-CAHPS study. J Geriatr Oncol. april 2021;12(3):446–52.

81. Mosallam RA, Metwally S. Patients' views on the quality of transitional care at a health insurance hospital in Alexandria, Egypt. J Egypt Public Health Assoc. august 2014;89(2):74–80.

82. Ning X, Krishnan A, Li X, Liu Z, Li J, Dai X, m.fl. Perceived quality of care and its associated factors among Chinese patients with advanced cancer: findings from the APPROACH study in Beijing. Support Care Cancer Off J Multinatl Assoc Support Care Cancer. 2021;29(3):1395–401.

83. Noel PH, Barnard JM, Barry FM, Simon A, Lee ML, Olmos-Ochoa TT, m.fl. Patient experience of health care system hassles: Dual-system vs single-system users. Health Serv Res. 2020;55(4):548–55.

84. O'Brien I, Britton E, Sarfati D, Naylor W, Borman B, Ellison-Loschmann L, m.fl. The voice of experience: results from Cancer Control New Zealand's first national cancer care survey. N Z Med J. 2010;123(1325):10–9.

85. O'Malley AS, Peikes D, Wilson C, Gaddes R, Peebles V, Day TJ, m.fl. Patients' perspectives of care management: a qualitative study. Am J Manag Care. 2017;23(11):684–9.

86. Penm J, MacKinnon NJ, Strakowski SM, Ying J, Doty MM. Minding the Gap: Factors Associated With Primary Care Coordination of Adults in 11 Countries. Ann Fam Med. 2017;15(2):113–9.

87. Pineault R, Provost S, Hamel M, Couture A, Levesque JF. The influence of primary health care organizational models on patients' experience of care in different chronic disease situations. Chronic Dis Inj Can. 2011;31(3):109–20.

88. Riley DL, Stewart DE, Grace SL. Continuity of cardiac care: Cardiac rehabilitation participation and other correlates. Int J Cardiol. juli 2007;119(3):326–33.

89. Ruud T, Aarre TF, Boeskov B, le Husevag PS, Klepp R, Kristiansen SA, m.fl. Satisfaction with primary care and mental health care among individuals with severe mental illness in a rural area: a seven-year follow-up study of a clinical cohort. Int J Ment Health Syst. 2016;10(101294224):33.

90. Scaioli G, Schäfer WLA, Boerma WGW, Spreeuwenberg P, van den Berg M, Schellevis FG, m.fl. Patients' perception of communication at the interface between primary and secondary care: a cross-sectional survey in 34 countries. BMC Health Serv Res. 30. december 2019;19(1):1018.

91. Schattner A, Bronstein A, Jellin N. Information and shared decision-making are top patients' priorities. BMC Health Serv Res. 2006;6(101088677):21.

92. Schoen C, Osborn R, Huynh PT, Doty M, Zapert K, Peugh J, m.fl. Taking the pulse of health care systems: experiences of patients with health problems in six countries. Health Aff Proj Hope. 2005;Suppl Web Exclusives(8303128, gag):W5-25.

93. Sequist TD, Von Glahn T, Li A, Rogers WH, Safran DG. Measuring chronic care delivery: patient experiences and clinical performance. Int J Qual Health Care. 1. juni 2012;24(3):206–13.

94. Sisler JJ, Taylor-Brown J, Nugent Z, Bell D, Khawaja M, Czaykowski P, m.fl. Continuity of care of colorectal cancer survivors at the end of treatment: the oncology–primary care interface. J Cancer Surviv. december 2012;6(4):468–75.

95. Stentebjerg Petersen G, Knudsen JL, Ejlersen E. [Cancer patients' experiences contribute to more patient-centred care]. Ugeskr Laeger. 2015;177(34):1616–9.

96. Sweeney A, Rose D, Clement S, Jichi F, Jones IR, Burns T, m.fl. Understanding service user-defined continuity of care and its relationship to health and social measures: a cross-sectional study. BMC Health Serv Res. 8. juni 2012;12:145.

97. Tremblay D, Roberge D, Touati N, Maunsell E, Berbiche D. Effects of interdisciplinary teamwork on patient-reported experience of cancer care. BMC Health Serv Res. december 2017;17(1):218.

98. Van Roij J, Raijmakers N, Ham L, Van Den Beuken-van Everdingen M, Van Den Borne B, Creemers GJ, m.fl. Quality of life and quality of care as experienced by patients with advanced cancer and their relatives: A multicentre observational cohort study (eQuiPe). Eur J Cancer. april 2022;165:125–35.

99. Vargas I, Waibel S, Vazquez ML, Aller MB, Coderch J, Sanchez-Perez I, m.fl. A comprehensive analysis of patients' perceptions of continuity of care and their associated factors. Int J Qual Health Care. 2013;25(3):291–9.

100. Viet-Thi Tran, Diard E, Ravaud P. Priorities to improve the care for chronic conditions and multimorbidity: a survey of patients and stakeholders nested within the ComPaRe e- cohort. BMJ Qual Saf. 2021;30(7):577–87.

101. Weinberg DB, Gittell JH, Lusenhop RW, Kautz CM, Wright J. Beyond our walls: impact of patient and provider coordination across the continuum on outcomes for surgical patients. Health Serv Res. 2007;42(1 Pt 1):7–24.

102. Wells R, Breckenridge ED, Siañez M, Tamayo L, Kum HC, Ohsfeldt RL. Self-Reported Quality, Health, and Cost-Related Outcomes of Care Coordination Among Patients with Complex Health Needs. Popul Health Manag. 1. februar 2020;23(1):59–67.

103. Willems LM, Kwakkenbos L, Bode C, van den Hoogen FHJ, van den Ende CHM. Health care use and patients' perceptions on quality of care in systemic sclerosis. Clin Exp Rheumatol. 2013;31(2 Suppl 76):64–70.

104. Williams KE, Sansoni J, Morris D, Thompson C. A Delphi study to develop indicators of cancer patient experience for quality improvement. Support Care Cancer. januar 2018;26(1):129–38.

105. Berbee R, Steuten LMG, Vrijhoef HJM, Wagner EH. Quality of integrated chronic care measured by patient survey: Identification, selection and application of most appropriate instruments. Health Expect. 2009;12(4):417–29.

106. Black D, Held ML, Skeesick J, Peters T. Measures Evaluating Patient Satisfaction in Integrated Health Care Settings: A Systematic Review. Community Ment Health J. november 2021;57(8):1464–77.

107. Fernandes S, Fond G, Zendjidjian XY, Baumstarck K, Lançon C, Berna F, m.fl. Measuring the Patient Experience of Mental Health Care: A Systematic and Critical Review of Patient-Reported Experience Measures. Patient Prefer Adherence. november 2020;Volume 14:2147–61.

108. Fillion L, Cook S, Veillette AM, Aubin M, de Serres M, Rainville F, m.fl. Professional Navigation Framework: Elaboration and Validation in a Canadian Context. Oncol Nurs Forum. 1. januar 2012;39(1):E58–69.

109. Fiscella K, Ransom S, Jean-Pierre P, Cella D, Stein K, Bauer JE, m.fl. Patient-reported outcome measures suitable to assessment of patient navigation. Cancer. august 2011;117(15 Suppl):3603–17.

110. McMurray J, McNeil H, Lafortune C, Black S, Prorok J, Stolee P. Measuring Patients' Experience of Rehabilitation Services Across the Care Continuum. Part I: A Systematic Review of the Literature. Arch Phys Med Rehabil. januar 2016;97(1):104–20.

111. Perriman N, Davis D. Measuring maternal satisfaction with maternity care: A systematic integrative review. Women Birth. juni 2016;29(3):293–9.

112. Quinn M, Robinson C, Forman J, Krein SL, Rosland AM. Survey Instruments to Assess Patient Experiences With Access and Coordination Across Health Care Settings: Available and Needed Measures. Med Care. 2017;55(Supplement 7):S84–91.

113. Schick-Makaroff K, Karimi-Dehkordi M, Cuthbertson L, Dixon D, Cohen SR, Hilliard N, m.fl. Using Patient- and Family-Reported Outcome and Experience Measures Across Transitions of Care for Frail Older Adults Living at Home: A Meta-Narrative Synthesis. The Gerontologist. 16. januar 2020;61(3):e23–38.

114. Volakakis N, Pylli M, Raftopoulos V, Kyrkou I, Xanthos T, Deltsidou A. Exploration of the factors that influence perceived quality of patient centered care among cancer survivors: A systematic review. Eur J Oncol Nurs. februar 2024;68:102503.

115. Weaver N, Coffey M, Hewitt J. Concepts, models and measurement of continuity of care in mental health services: A systematic appraisal of the literature. J Psychiatr Ment Health Nurs. august 2017;24(6):431–50.

116. Yoshimura M, Sumi N. Measurement tools that assess the quality of transitional care from patients' perspective: A literature review. Jpn J Nurs Sci. 2022;19(3):e12472.

117. Acosta AM, Lima MADS, Marques GQ, Levandovski PF, Weber LAF. Brazilian version of the Care Transitions Measure: translation and validation. Int Nurs Rev. 2017;64(3):379–87.

118. Aller MB, Vargas I, Garcia I, Coderch J, Colomés L, Llopart JR, m.fl. A tool for assessing continuity of care across care levels: an extended psychometric validation of the CCAENA questionnaire. Int J Integr Care [Internet]. 2. december 2013 [henvist 2. juni 2023];13(4). Tilgængelig hos: http://www.ijic.org/article/10.5334/ijic.1160/

119. Badri MA, Attia ST, Ustadi AM. Testing not-so-obvious models of healthcare quality. Int J Health Care Qual Assur. 2008;21(2):159–74.

120. Bakshi AB, Wee SL, Tay C, Wong LM, Leong IYO, Merchant RA, m.fl. Validation of the care transition measure in multi-ethnic South-East Asia in Singapore. BMC Health Serv Res. 2012;12(101088677):256.

121. Bentler SE, Morgan RO, Virnig BA, Wolinsky FD. Do claims-based continuity of care measures reflect the patient perspective?. Med Care Res Rev MCRR. 2014;71(2):156–73.

122. Bentler SE, Morgan RO, Virnig BA, Wolinsky FD. Evaluation of a patient-reported continuity of care model for older adults. Qual Life Res Int J Qual Life Asp Treat Care Rehabil. 2014;23(1):185–93.

123. Berendsen AJ, Groenier KH, de Jong GM, Meyboom-de Jong B, van der Veen WJ, Dekker J, m.fl. Assessment of patient's experiences across the interface between primary and secondary care: Consumer Quality Index Continuum of care. Patient Educ Couns. 2009;77(1):123–7.

124. Boyer L, Fernandes S, Brousse Y, Zendjidjian X, Cano D, Riedberger J, m.fl. Development of the PREMIUM computerized adaptive testing for measuring the access and care coordination for patients with severe mental illness. Psychiatry Res. oktober 2023;328:115444.

125. Bull MJ, Luo D, Maruyama GM. Measuring continuity of elders' posthospital care. J Nurs Meas. 2000;8(1):41–60.

126. Castle N, Engberg J, Men A. Satisfaction of Discharged Nursing Home Residents. J Appl Gerontol Off J South Gerontol Soc. 2018;37(10):1225–43.

127. Chavez LM, Canino G, Shrout PE, Barrio C, Ware NC. Psychometric evaluation of the Spanish version of CONNECT: a measure of continuity of care in mental health services. Int J Methods Psychiatr Res. 2007;16(1):23–33.

128. Clark K, Beatty S, Reibel T. Maternity-care: measuring women's perceptions. Int J Health Care Qual Assur. 2016;29(1):89–99.

129. Cohen Castel O, Dagan E, Keinan-Boker L, Shadmi E. Reliability and validity of the Hebrew version of the Nijmegen Continuity Questionnaire for measuring patients' perceived continuity of care in oral anticancer therapy. Eur J Cancer Care (Engl). 2018;27(6):e12913.

130. Coleman EA, Mahoney E, Parry C. Assessing the quality of preparation for posthospital care from the patient's perspective: the care transitions measure. Med Care. 2005;43(3):246–55.

131. Crump H, King J, Graham C, Thorlby R, Raleigh V, Redding D, m.fl. Developing a User Reported Measure of Care Co-ordination. Int J Integr Care. 2017;17(1):4.

132. Dolovich LR, Nair KM, Ciliska DK, Lee HN, Birch S, Gafni A, m.fl. The Diabetes Continuity of Care Scale: the development and initial evaluation of a questionnaire that measures continuity of care from the patient perspective. Health Soc Care Community. 2004;12(6):475–87.

133. Drewes HW, de Jong-van Til JT, Struijs JN, Baan CA, Tekle FB, Meijboom BR, m.fl. Measuring chronic care management experience of patients with diabetes: PACIC and PACIC+ validation. Int J Integr Care. 2012;12(101214424):e194.

134. Eubank BH, Lafave MR, Mohtadi NG, Sheps DM, Wiley JP. Validation of a tool to assess patient satisfaction, waiting times, healthcare utilization, and cost. Prim Health Care Res Dev. 2019;20(100897390):e47.

135. Fulton BR, Sternke EA, Ayala L, Malott DLJ. Psychometric Testing of a Measure of Patient Experience in an Ambulatory Surgery Setting. J Ambulatory Care Manage. 2019;42(1):27–36.

136. Glasgow RE, Wagner EH, Schaefer J, Mahoney LD, Reid RJ, Greene SM. Development and validation of the Patient Assessment of Chronic Illness Care (PACIC). Med Care. maj 2005;43(5):436–44.

137. Graumlich JF, Novotny NL, Aldag JC. Brief scale measuring patient preparedness for hospital discharge to home: Psychometric properties. J Hosp Med. 2008;3(6):446–54.

138. Gulliford MC, Naithani S, Morgan M. Measuring continuity of care in diabetes mellitus: an experience-based measure. Ann Fam Med. 2006;4(6):548–55.

139. Hadjistavropoulos HD, Biem HJ, Kowalyk KM. Measurement of continuity of care in cardiac patients: reliability and validity of an in-person questionnaire. Can J Cardiol. 2004;20(9):883–91.

140. Hadjistavropoulos H, Sharpe D, Bourgault-Fagnou M, Janzen J, Biem H. Patient perceptions of hospital discharge: Reliability and validity of a patient continuity of care questionnaire. Int J Qual Health Care. 2008;20(5):314–23.

141. Haggerty JL, Burge F, Pineault R, Beaulieu MD, Bouharaoui F, Beaulieu C, m.fl. Management continuity from the patient perspective: comparison of primary healthcare evaluation instruments. Healthc Policy Polit Sante. 2011;7(Spec Issue):139–53.

142. Haggerty JL, Roberge D, Freeman GK, Beaulieu C, Bréton M. Validation of a generic measure of continuity of care: when patients encounter several clinicians. Ann Fam Med. 2012;10(5):443–51.

143. Hennrich P, Arnold C, Koetsenruijter J, Wensing M. Measuring continuity of ambulatory cardiovascular care: a cross-sectional study on the applicability of the Nijmegen Continuity Questionnaire in Germany. BMC Health Serv Res. 18. oktober 2022;22(1):1258.

144. Hetlevik Ø, Hustoft M, Uijen A, Aßmus J, Gjesdal S. Patient perspectives on continuity of care: adaption and preliminary psychometric assessment of a Norwegian version of the Nijmegen Continuity Questionnaire (NCQ-N). BMC Health Serv Res. 21. november 2017;17(1):760.

145. Husain A, Barbera L, Howell D, Moineddin R, Bezjak A, Sussman J. Advanced lung cancer patients' experience with continuity of care and supportive care needs. Support Care Cancer Off J Multinatl Assoc Support Care Cancer. 2013;21(5):1351–8.

146. Hwang JI, Chung JH, Kim HK. Psychometric properties of transitional care instruments and their relationships with health literacy: Brief PREPARED and Care Transitions Measure. Int J Qual Health Care J Int Soc Qual Health Care. 31. december 2019;31(10):774–80.

147. Ignatyev Y, Timm J, Heinze M, Indefrey S, von Peter S. Development and Preliminary Validation of the Scale for Evaluation of Psychiatric Integrative and Continuous Care-Patient's Version. Front Psychiatry. 2017;8(101545006):162.

148. Sollid MIV, Slaaen M, Danielsen S, Kirkevold Ø. Psychometric properties of the person-centred coordinated care experience questionnaire (P3CEQ) in a Norwegian radiotherapy setting. Int J Qual Health Care J Int Soc Qual Health Care. 15. september 2022;34(3):mzac067.

149. Joober H, Chouinard MC, King J, Lambert M, Hudon E, Hudon C. The Patient Experience of Integrated Care Scale: A Validation Study among Patients with Chronic Conditions Seen in Primary Care. Int J Integr Care. 2018;18(4):1.

150. Joyce AS, Adair CE, Wild TC, McDougall GM, Gordon A, Costigan N, m.fl. Continuity of care: validation of a self-report measure to assess client perceptions of mental health service delivery. Community Ment Health J. april 2010;46(2):192–208.

151. Kiang MV, Singer SJ, Friedberg MW, Dunn T, Kuhn DM. Development and preliminary validation of the Patient Perceptions of Integrated Care survey. Med Care Res Rev. 2013;70(2):143–64.

152. Kim SK, Hwang YS, Ock M, Jo HS. Development of Items for Transitional Care Service and Outcome Indicators of Discharged Patients for Improvement in Quality of Care. J Korean Med Sci. 2023;38(32):e246.

153. Kowalyk KM, Hadjistavropoulos HD, Biem HJ. Measuring continuity of care for cardiac patients: development of a patient self-report questionnaire. Can J Cardiol. 2004;20(2):205–12.

154. Liu LM, Liu MT, Sun MJ, Wang JN, Lin BL, Wang P, m.fl. Validity and reliability of the Chinese version of the partners at care transitions measure. BMC Health Serv Res. 2021;21(1):1284.

155. Ljungholm L, Årestedt K, Fagerström C, Djukanovic I, Ekstedt M. Measuring patients' experiences of continuity of care in a primary care context-Development and evaluation of a patient-reported experience measure. J Adv Nurs. januar 2024;80(1):387–98.

156. Lloyd H, Fosh B, Whalley B, Byng R, Close J. Validation of the person-centred coordinated care experience questionnaire (P3CEQ). Int J Qual Health Care J Int Soc Qual Health Care. 2019;31(7):506–12.

157. Malik N, Alvaro C, Kuluski K, Wilkinson AJ. Measuring patient satisfaction in complex continuing care/rehabilitation care. Int J Health Care Qual Assur. 2016;29(3):324–36.

158. Masters S, Giles L, Halbert J, Crotty M. Development and testing of a questionnaire to measure older people's experience of the Transition Care Program in Australia. Australas J Ageing. 2010;29(4):172–8.

159. McAlister FA, Lin M, Bakal J, Kemp KA, Quan H. The Care Transitions Measure-3 Is Only Weakly Associated with Post-discharge Outcomes: a Retrospective Cohort Study in 48,384 Albertans. J Gen Intern Med. 2019;34(11):2497–504.

160. McGuiness C, Sibthorpe B. Development and initial validation of a measure of coordination of health care. Int J Qual Health Care J Int Soc Qual Health Care. august 2003;15(4):309–18.

161. Mira JJ, Nuno-Solinis R, Guilabert-Mora M, Solas-Gaspar O, Fernandez-Cano P, Gonzalez-Mestre MA, m.fl. Development and Validation of an Instrument for Assessing Patient Experience of Chronic Illness Care. Int J Integr Care. 2016;16(3):13.

162. Noest S, Ludt S, Klingenberg A, Glassen K, Heiss F, Ose D, m.fl. Involving patients in detecting quality gaps in a fragmented healthcare system: development of a questionnaire for Patients' Experiences Across Health Care Sectors (PEACS). Int J Qual Health Care. 1. juni 2014;26(3):240–9.

163. Oikonomou E, Page B, Lawton R, Murray J, Higham H, Vincent C. Validation of the Partners at Care Transitions Measure (PACT-M): assessing the quality and safety of care transitions for older people in the UK. BMC Health Serv Res. 2020;20(1):608.

164. Parra-Vega I, Marques-Sanchez P, Pelayo-Teran JM, Corral Gudino L. Development and validation of a questionnaire for assessing patients' perceptions of interprofessional integration in health care. J Interprof Care. 2021;(9205811):1–7.

165. Peabody M, Bradley KD, Custer M. Assessing the Validity of a Continuum-of-care Survey: A Rasch Measurement Approach. J Appl Meas. 2016;17(1):1–13.

166. Radwin LE, Cabral HJ, Seibert MN, Stolzmann K, Meterko M, Evans L, m.fl. Patient-Centered Care in Primary Care Scale: Pilot Development and Psychometric Assessment. J Nurs Care Qual. 2019;34(1):34–9.

167. Ramond-Roquin A, Stewart M, Ryan BL, Richards M, Sussman J, Brown JB, m.fl. The "Patient-centered coordination by a care team" questionnaire achieves satisfactory validity and reliability. J Interprof Care. 2019;33(5):558–69.

168. REYNOLDS J, GADSBY E, RIJKEN M, STOOP A, ESPALLARGUES M, LLOYD HM, m.fl. Measuring Older Peoples' Experiences of Person-Centred Coordinated Care: Experience and Methodological Reflections from Applying a Patient Reported Experience Measure in SUSTAIN. Int J Integr Care IJIC. 2021;21(3):1–17.

169. Rijken M, Close J, Menting J, Lette M, Stoop A, Zonneveld N, m.fl. Assessing the experience of person-centred coordinated care of people with chronic conditions in the Netherlands: Validation of the Dutch P3CEQ. Health Expect. juni 2022;25(3):1069–80.

170. Rose D, Sweeney A, Leese M, Clement S, Jones IR, Burns T, m.fl. Developing a user-generated measure of continuity of care: brief report. Acta Psychiatr Scand. april 2009;119(4):320–4.

171. Rosenlund L, Jakobsson S, Lloyd H, Lundgren-Nilsson Å, Hermansson M, Dencker A. Measuring patient experiences of person-centred care: Translation, cultural adaption and qualitative evaluation of item candidates for use in England and Sweden. Scand J Caring Sci. marts 2022;36(1):235–44.

172. Rucci P, Foglino S, Bravi F, D'Avenia R, Altini M, Carradori T, m.fl. Validation of the OPportunity for Treatment In ONcology (OPTION) questionnaire measuring continuity of care. Eur J Cancer Care (Engl). 2018;27(1).

173. Seo AR, Kim BK, Park KS. Psychometric Properties and Effects on Health Outcomes of the Patient Assessment of Chronic Illness Care (PACIC) in Korean Hemodialysis Patients. Healthcare. 20. juni 2022;10(6):1149.

174. Shadmi E, Zisberg A, Coleman EA. Translation and validation of the Care Transition Measure into Hebrew and Arabic. Int J Qual Health Care J Int Soc Qual Health Care. 2009;21(2):97–102.

175. Smith LFP. Postnatal care: development of a psychometric multidimensional satisfaction questionnaire (the WOMBPNSQ) to assess women's views. Br J Gen Pract J R Coll Gen Pract. 2011;61(591):e628-37.

176. Soares JB, Nogueira MC, Fernandes D, Goncalves BM, Goncalves R. Validation of the Portuguese version of a questionnaire to measure Quality of Care Through the Eyes of Patients with Inflammatory Bowel Disease (QUOTE-IBD). Eur J Gastroenterol Hepatol. 2015;27(12):1409–17.

177. Sorra J, Zebrak K, Carpenter D, Famolaro T, Rauch J, Li J, m.fl. Development and psychometric properties of surveys to assess patient and family caregiver experience with care transitions. BMC Health Serv Res. 2021;21(1):785.

178. Squitieri L, Tsangaris E, Klassen AF, van Haren ELWG, Poulsen L, Longmire NM, m.fl. Patient-reported experience measures are essential to improving quality of care for chronic wounds: An international qualitative study. Int Wound J. 2020;17(4):1052–61.

179. Sugavanam T, Fosh B, Close J, Byng R, Horrell J, Lloyd H. Codesigning a Measure of Person-Centred Coordinated Care to Capture the Experience of the Patient: The Development of the P3CEQ. J Patient Exp. 2018;5(3):201–11.

180. Teale EA, Young JB. A Patient Reported Experience Measure (PREM) for use by older people in community services. Age Ageing. 2015;44(4):667–72.

181. Thompson R, Stevens G, Elwyn G. Measuring Patient Experiences of Integration in Health Care Delivery: Psychometric Validation of IntegRATE Under Controlled Conditions. J Patient Exp. 2021;8(101688338):23743735211007346.

182. Uijen A, Schellevis F, Bosch W, Mokkink H, Weel C, Schers H. Nijmegen Continuity Questionnaire: Development and testing of a questionnaire that measures continuity of care. J Clin Epidemiol. 18. juni 2011;64:1391–9.

183. Uijen AA, Schers HJ, Schellevis FG, Mokkink HGA, van Weel C, van den Bosch WJ. Measuring continuity of care: psychometric properties of the Nijmegen Continuity Questionnaire. Br J Gen Pract J R Coll Gen Pract. 2012;62(600):e949-57.

184. Valaker I, Fridlund B, Wentzel-Larsen T, Hadjistavropoulos H, Nordrehaug JE, Rotevatn S, m.fl. Adaptation and psychometric properties of the Norwegian version of the heart continuity of care questionnaire (HCCQ). BMC Med Res Methodol. 2019;19(1):62.

185. van Melle MA, van Stel HF, Poldervaart JM, de Wit NJ, Zwart DLM. The transitional risk and incident questionnaire was valid and reliable for measuring transitional patient safety from the patients' perspective. J Clin Epidemiol. 2019;105(jce, 8801383):40–9.

186. Vimalananda VG, Meterko M, Sitter KE, Qian S, Wormwood JB, Fincke BG. Patients' Experience of Specialty Care Coordination: Survey Development and Validation. J Patient-Centered Res Rev. 27. november 2023;10(4):219–30.

187. Walker KO, Stewart AL, Grumbach K. Development of a survey instrument to measure patient experience of integrated care. BMC Health Serv Res. 2016;16(101088677):193.

188. Ware NC, Dickey B, Tugenberg T, McHorney CA. CONNECT: A Measure of Continuity of Care in Mental Health Services. Ment Health Serv Res. 1. december 2003;5(4):209–21.

189. Young JM, Walsh J, Butow PN, Solomon MJ, Shaw J. Measuring cancer care coordination: development and validation of a questionnaire for patients. BMC Cancer. 2011;11(100967800):298.
